# Amino Acid Permeases and Virulence in *Cryptococcus neoformans*

**DOI:** 10.1371/journal.pone.0163919

**Published:** 2016-10-03

**Authors:** Kevin Felipe Cruz Martho, Amanda Teixeira de Melo, Juliana Possato Fernandes Takahashi, Juliana Mariotti Guerra, Dayane Cristina da Silva Santos, Sônia Ueda Purisco, Márcia de Souza Carvalho Melhem, Raquel dos Anjos Fazioli, Clerlune Phanord, Patrícia Sartorelli, Marcelo A. Vallim, Renata C. Pascon

**Affiliations:** 1 Instituto de Ciências Ambientais, Química e Farmacêuticas, Universidade Federal de São Paulo, Rua Arthur Ridel, 275, Diadema, SP, Brazil; 2 Quantitative Pathology Unit, Adolfo Lutz Institute, São Paulo, Brazil; 3 Mycology Unit, Adolfo Lutz Institute, Secretary of Health, São Paulo, Brazil; Yonsei University, REPUBLIC OF KOREA

## Abstract

Fungal opportunistic pathogens colonize various environments, from plants and wood to human and animal tissue. Regarding human pathogens, one great challenge during contrasting niche occupation is the adaptation to different conditions, such as temperature, osmolarity, salinity, pressure, oxidative stress and nutritional availability, which may constitute sources of stress that need to be tolerated and overcome. As an opportunistic pathogen, *C*. *neoformans* faces exactly these situations during the transition from the environment to the human host, encountering nutritional constraints. Our previous and current research on amino acid biosynthetic pathways indicates that amino acid permeases are regulated by the presence of the amino acids, nitrogen and temperature. *Saccharomyces cerevisiae* and *Candida albicans* have twenty-four and twenty-seven genes encoding amino acid permeases, respectively; conversely, they are scarce in number in Basidiomycetes (*C*. *neoformans*, *Coprinopsis cinerea* and *Ustilago maydis*), where nine to ten permease genes can be found depending on the species. In this study, we have demonstrated that two amino acid permeases are essential for virulence in *C*. *neoformans*. Our data showed that *C*. *neoformans* uses two global and redundant amino acid permeases, Aap4 and Aap5 to respond correctly to thermal and oxidative stress. Double deletion of these permeases causes growth arrest in *C*. *neoformans* at 37°C and in the presence of hydrogen peroxide. The inability to uptake amino acid at a higher temperature and under oxidative stress also led to virulence attenuation *in vivo*. Our data showed that thermosensitivity caused by the lack of permeases Aap4 and Aap5 can be remedied by alkaline conditions (higher pH) and salinity. Permeases Aap4 and Aap5 are also required during fluconazole stress and they are the target of the plant secondary metabolite eugenol, a potent antifungal inhibitor that targets amino acid permeases. In summary, our work unravels (i) interesting physiological property of *C*. *neoformans* regarding its amino acid uptake system; (ii) an important aspect of virulence, which is the need for amino acid permeases during thermal and oxidative stress resistance and, hence, host invasion and colonization; and (iii) provides a convenient prototype for antifungal development, which are the amino acid permeases Aap4/Aap5 and their inhibitor.

## Introduction

Microorganisms are highly exposed to environmental changes. The quick response to unstable conditions may be decisive to their survival [1]. Fungal pathogens typically face this challenge since they are encountered as free living organisms inhabiting the natural environment, but they can also invade animal hosts, which pose a completely different condition. Temperature, osmolarity, oxidative stress, pressure and nutritional status are among the environmental parameters that require rapid and efficient adaptation [2–8].

Opportunistic yeasts are a good example of pathogens that often need to cope with this situation during their life cycle. That is the case of the basidiomycete *C*. *neoformans*, which is frequently found on decomposed wood, pigeon guano and soil [9–11]. However, it can also infect and cause disease in animals, such as cats, goats, koalas [9,11]. In humans, *C*. *neoformans* causes fungal meningoencephalitis, a disease that is very common among immunocompromised patients due to AIDS, organ transplantation and those undergoing chemotherapy [12–14]. The course of the disease starts when spores or desiccated yeast cells are inhaled and can establish pulmonary infection, which can be cleared in an immunocompetent host or progress to fungal meningitis, leading to a serious condition in immunocompromised patients, [10,15]. Therefore, cryptococcosis is one of the most important causes of mortality/morbidity in HIV/AIDS patients, with an estimated 624.700 deaths/year, mainly in sub-Saharan Africa [16].

In order to invade, survive and colonize the host, *C*. *neoformans* has key virulence factors (capsule, melanine, phospholipase, urease and thermo-tolerance) that have been extensively described in the literature and enable it to fight the host defense mechanisms [9–11,15,17,18]. Furthermore, *C*. *neoformans* is able to respond rapidly and efficiently to adverse conditions, such as high temperature, oxidative, osmotic, alkaline and nutritional stress. This ability guarantees survival and colonization and, therefore, is an important component of the pathogenesis [2,3,6,19–26]

Among several nutrients that are important for survival in different environments, amino acids are largely known as a relevant and their biosynthesis has been considered as excellent targets for novel antifungal drugs [27–34]. Among several biosynthetic routes studied in the past 15 years, Kingsbury and McCusker (2008) showed that the threonine biosynthetic pathway is essential. Recently, our group demonstrated that tryptophan biosynthesis is also essential in *C*. *neoformans* and several compounds inhibit growth by directly interfering with key enzymes of this pathway, thus revealing the potential application of this research [27]. In addition, the lethality, caused by perturbations on these pathways, is quite unusual in fungi, since amino acid uptake by permeases can often remediate auxotrophs. Lethality may be due to the small number of amino acid permeases encoded by the *C*. *neoformans* genome and also, it might be attributed to the low enzyme-substrate affinity, which seems to be dependent upon temperature, as previously pointed out by our research and others [27,28].

The genome organization of the amino acid permeases in *C*. *neoformans* is a rather peculiar one: bioinformatics tools revealed a total of ten cytoplasm permease genes, of which, eight seem to encode global permeases (*AAP*1 to *AAP*8) and two are similar to *S*. *cerevisiae* Mup1 and Mup3 sulfur amino acid permeases [27]. All of them belong to the APC (Amino acid–Polyamine-Choline transporter). This superfamily is wide spread among bacteria, archaea, yeasts, fungi, unicellular eukaryotic protists, slime molds, plants and animals [35], the transporters are solute:cation symporters and solute:solute antiporters and several of them have twelve transmembrane α-helical spanners [36]. Interestingly, this genomic organization of the permeases seems to be shared by other Basidiomycetes, since the same configuration was observed in *U*. *maydis* and *C*. *cinerea*. Other yeasts, such as *C*. *albicans* and *S*. *cerevisiae*, and filamentous ascomycetes, as *Aspergillus nidulans*, encode a broader range of permeases [27].

In *S*. *cerevisiae*, the twenty-four amino acid permeases of the APC-superfamily are divided into four clusters according to the biochemical features of the amino acids they transport and whether they have a broad or narrow affinity for the substrates [37]. In a general sense, there are three broad permeases (Agp1, Gap1 and Agp3) that vary in transport capacity (low to high) and the remaining permeases have a low or high affinity to specific amino acids or groups of related amino acids, according to their side chain [37–40]. Permease expression is under the control of at least three major regulatory mechanisms: Nitrogen Catabolite Repression (NCR), Global Amino Acid Control (GAAC), and the SPS-sensing system. These three mechanisms respond to nitrogen quality, amino acid starvation and the presence of amino acids in the extracellular compartment, respectively [38,41,42].

Recently, six homologues of *S*. *cerevisiae* Gap1 have been identified in *C*. *albicans* and shown to act not only as transporters, but also as sensors via PKA [43]. There is mounting evidence that amino acid permeases are important for virulence in *C*. *albicans* [44–46]. Along these lines, *Trypanosoma brucei* low-selective/low-affinity neutral amino acid transporter (AAT6) has been explored as a putative pharmacological target [47]. Similarly, a monospecific proline transporter (AAAP069) of *Trypanosoma cruzei*, the etiological agent of Chagas disease, has been correlated to drug resistance, hydrogen peroxide and nitric oxide stress [48]. In *S*. *cerevisiae*, Gap1 and Tat2 are the targets of eugenol, a plant secondary metabolite that inhibits a broad range of microbes [49].

In spite of the previous description of the *C*. *neoformans* amino acid permease published by our group, there is as yet no information on how this uptake system operates in opportunistic yeasts and on basidiomycetes in general. Furthermore, we do not know whether amino acid permeases would play a role in virulence or whether they would be required for *in vitro* and *in vivo* survival in *C*. *neoformans*.

In the current study, we investigated five amino acid permeases of *C*. *neoformans* in order to identify their substrate specificity and their role in growth and virulence. These five genes (*AAP*2, *AAP*4, *AAP*5, *MUP*1 and *MUP*3) were chosen based on the (i) expression pattern induced by the nitrogen source (NCR), starvation and extracellular amino acids (*AAP*2, *AAP*4 and *AAP*5), (ii) redundancy (*AAP*4 and *AAP*5) and (iii) specificity inferred by bioinformatics studies (*MUP*1 and *MUP*3). In this work, permease regulatory mechanisms were further studied and the role of temperature on their expression was established by qPCR and mutant analysis. Single and double gene deletion allowed us to identify two major players among the permeases and, from those, infer their specificity, mode of action and role in survival both *in vitro* and *in vivo*. Our study underlined an overlap between amino acid uptake, high-temperature growth, oxidative stress response and virulence. Finally, we demonstrated that amino acid permeases play a role in fluconazole resistance and are the target of plant secondary metabolite with antifungal properties. Our results indicate that amino acid permeases are important player in fungal pathogenesis and suggest it as a valuable drug target for the discovery and development of inhibitors.

## Materials and Methods

### Strains and media

The strains used are list in S1 Table. All stains in this study were derived of the serotype A H99 wild type (WT) *C*. *neoformans* var. *grubii*. The strains were grown routinely on rich medium YPD (1% yeast extract, 2% bacto-peptone, 2% glucose); synthetic dextrose (SD) was prepared with yeast nitrogen base, YNB (0.67g/L yeast nitrogen base w/o amino acid and ammonium sulfate, 2% glucose, 10mM nitrogen source) unless specified otherwise, at 30°C or 37°C with 150 rpm in a rotary shaker. YPG was prepared with 2% of galactose instead of dextrose. Spot dilutions were made by growing overnight cultures in YEPD, cells were washed twice in sterile saline, adjusted to 2 x 10^6^ CFU/mL and serial diluted until to 2 x 10^2^ CFU/mL. Five microliters of each dilution were spotted on test plates.

### Genetic manipulations

Permeases deletion mutants were create by substitution of the coding region (start to stop) by a selectable marker (resistance to hygromycin or G418). Constructs were generated by double-joint PCR method as described elsewhere [50]. All primers used to generate the construct for gene deletion are listed in S2 Table. Deletion constructs were introduced into wild type H99 strain by biolistic transformation [51]. Transformants were selected on YPD plates supplemented with 200 μg/mL of either G418 or Hygromicin. In order to generate double mutants the *aap*4Δ::*Hph*^R^ construct was introduced into *aap*5Δ::*Neo*^R^ strain (CNU050) and *mup*1Δ::*Hph*^R^ construct into *mup*3Δ::*Neo*^R^ strain (CNU079). Colonies resistant to Hygromicyn or G418 were selected and tested by diagnostic PCR and Southern blot was used to confirm the homologous integration of the deletion constructs (S1 Fig). At least two independent transformants were initially tested for each single and double deletion (S2 Table). All other genetic engineering techniques were done according to standard protocols published by Sambrook *et al*., (1989).

### Growth rate on amino acids

The ability to grow on amino acid as sole nitrogen source was evaluated on 96 well plates in 100 μL total volume of SD added with YNB, 2% dextrose and 10 mM of a single amino acid as sole nitrogen source, nineteen amino acids were tested. Cells were grown overnight in YEPD at 30°C, washed 3 times in sterile PBS. Intracellular nitrogen pools were exhausted by incubation of the washed cells in PBS (Phosphate Buffered Saline) at 30°C with 150 rpm rotation for 2 hours. After this period, a total of 200 cells were inoculated in each well containing a single amino acid as sole nitrogen source. All experiments were done in technical triplicates; plates were incubated at either 30 or 37°C for 48 hours. The OD_600_ was measured in a plate reader (Logen). A minimum of three biological replicates were done for all experiments. Assay controls: inoculums were cultivated on medium with either ammonium sulfate (positive control) or w/o it (negative control) in the same condition described above.

### *In vitro* virulence and stress resistance assays

In order to evaluate capsule production, the cells were cultivated in YPD medium at 30°C with orbital shaking (150 rpm) overnight, they were collected by centrifugation, washed three times with PBS 1X and normalized to an OD_600_ of 0.3 in CO_2_ independent medium (Gibco BRL) 1X and incubated at 30°C and 37°C for up to 72 hours [52]. Cells were stained with BactiDrop India Ink (Remel) for capsule observation under the light microscope. Capsules were documented at 24, 48 and 72 hours using MIPro Standard v1.1 Software. Quantitative analysis of capsule diameter was performed as described before [53]. Urease, phospholipase and melanine production were evaluated according the published protocol [8,24,54].

Osmotic stress was evaluated on YPD plates supplemented with KCl or NaCl (0.75 and 1 M) and alkaline stress by raising the pH to 6 and 7. Also, cell wall and plasma membrane integrity was evaluated in rich and synthetic medium supplemented with Congo red (0.5%) and SDS (0.03%), respectively.

Oxidative stress was evaluated on YEPD and SD (plus ammonium sulfate and ammonium sulfate plus amino acids) plates supplemented with hydrogen peroxide (1, 2.5 and 5 mM). All experiments were done at 30°C in triplicates.

### Antifungal sensibility test

Sensibility assays were done with E-test gradient strips of fluconazole and amphotericin B (Biomerieux). In brief: RPMI agar plates were inoculated with a cell suspension (OD_600_ = 0,25) using a sterile cotton swab, gradient strips were placed on the surface of the plate with sterile tweezers and plates were incubated at 30 and 37°C for 48 hours. Interpretation of the results was done according to the supplier’s manual. Minimum inhibitory concentration was determined according to the Clinical and Laboratory Standards Institute (CLSI M27-A2) with small adaptations describe before [27]. Extracted eugenol was diluted in 10% DMSO.

### Essential oil extraction and purification of eugenol

No specific permissions were required for plants collected to essential oil isolation (private property). The authors thank Ms. Célia Alem for plant material donation. Fresh leaves (300 g) of *Pimenta dioica*, a species collected at City of Rio Claro-SP, were extracted over 5 h by steam distillation in a Clevenger type apparatus to afford 1.54 g of crude essential oil. The oil was analyzed by GC-FID on a Shimadzu GC-2010 gas chromatograph equipped with an FID-detector and an automatic injector (Shimadzu AOC-20i) using a RtX-5 (5% phenyl, 95% polydimethylsiloxane (Restek, Bellefonte, PA, USA, 30 m × 0.32 mm × 0.25 μm film thickness) capillary column. These analyses were performed by injecting 1.0 μL of a 1.0 mg/mL solution of volatile oil in CH_2_Cl_2_ in a split mode (1:10) employing helium as the carrier gas (1 mL/min) under the following conditions: injector and detector temperatures of 220°C and 250°C, respectively; oven programmed temperature from 40–240°C at 3°C/min, holding 5 min at 240°C. GC-FID was performed in quantitative analysis. The major peak corresponded to 74% in the analysis by CG. Thus part of crude oil (1.5 g) was subjected to flash chromatography on SiO_2_ gel column (63 cm × 5 cm i.d.) chromatography eluted with CH_2_Cl_2_-methanol in proportions of (100:0, 99:1, 97:3, 95:5, 93:7, 80:20) (120 mL for each eluent) to afford 72 fractions which were individually analyzed using GC-FID and then pooled into thirteen groups (A to M). Fraction D was composed of pure eugenol (833.3 mg). The identification of eugenol was performed by comparing the data obtained from the spectral ^1^H and ^13^C NMR with the literature data [55].

### *In vivo* virulence assay

The assays with *Galleria mellonella* were conducted according to previous published protocol [56]. Isolated colonies were inoculated into 5 mL of rich medium and incubated with orbital agitation 150 rpm for 16-18h. Subsequently, suspensions were collected by centrifugation, washed twice in sterile PBS and adjusted to 1x10^6^cell/mL in PBS supplemented with ampicilin (20mg/kg body weight). Groups of 16 caterpillars with 200mg of average weight were inoculated with 10μL of the suspension with the aid of a Hamilton syringe in the region of the last pro-paw. Thereafter, caterpillars were separated on glass Petri dishes (15mm diameter) and incubated at 30°C and 37°C during 8 days. Caterpillars were monitored daily by observing spontaneous or provoked movements with the aid of a previously sterilized clip. The experiment was completed when the larvae die or formed cocoons.

Animal experimental protocols were reviewed and approved by the Committee de Ethics and Animal Use (CEUA) Instituto Adolfo Lutz/Pasteur. Outbred eight-week-old male BALB/c mice were purchased and maintained in IAL facilities (Instituto Aldolfo Lutz), provided with food and water *ad libitum*. Animal assay procedure was done by growing overnight cultures of yeast cells in Sabouraud dextrose broth medium (Becton Dickinson) at 30°C with moderate shaking (150 rpm) up to 24 h. Subsequently the obtained cultured cells were centrifuged, washed twice with PBS, cell were counted using a haemocytometer and adjusted to a desired concentration (2x10^5^ CFU/mL) in PBS. For concentration accuracy *C*. *neoformans* cell were platted on SDA and incubated at 30°C for 72 hours followed by counting the colony-forming units (CFU). For intranasal instillation, groups of 10 mice were anesthetized with 10 mg/kg ketamine (Syntec, Br) and 125 mg/kg xylazine (Syntec, Br) intraperitoneal, hung vertically from a surgical board, then 50μL of a cells suspension (2x10^6^ CFU/mL in PBS) was introduced into their nostrils by pipetting. Mice were monitored daily for 40 days. Animals that demonstrated weight loss (15% of initial weight) or clinical debilitation were cared and humanely euthanized with CO_2_ and every effort was made to minimize suffering. After 40 days post inoculation, mice that survived were euthanized with CO_2_ for fungal burden analysis. Lungs, brain, liver and spleen were excised and placed into separate tubes containing 4 mL sterile PBS on ice. Subsequently, the organs were homogenized using a sterilized tube and pestle attached to a mechanical tissue homogenizer (Potter N-136, Nova Técnica Br) and plated at various decreasing concentrations on SDA (Sabouread Dextrose Agar). Plates were incubated at 30^°^C for 72 h prior to enumeration of *C*. *neoformans* CFU.

### Histopathological analysis

Following euthanasia, right upper lobe of the lungs, spleen, liver and brain were preserved in 10% buffered formalin acetate (Fisher Scientific), embedded in paraffin; sectioned at 3μm and stained with Hematoxilin-Eosin (H&E) reagent. Images of the slides were acquired using an Olympus BX51 light microscope. Three Balb/C mice in each experimental and control group were analyzed. Vertebrate animal model was done with Balb/C.

### qPCR

Transcriptional pattern analysis was done from total RNA. In brief: the target strain was incubated overnight in liquid YPD under 150 rpm orbital shaking. The cells were harvested by centrifugation, washed twice in sterile ultrapure water and ressuspended in the required medium for 2 hours, which was previously equilibrated to the appropriate temperature (30 or 37°C). RNA extraction was conducted according to the previously published protocol [57] with slight modifications as follow: 3x10^8^ cells grown under the desired condition were centrifuged and ressuspended in 750 μL Trizol (Invitrogen) and same volume of glass beads (0.5 mm). The mixture was homogenized six to ten times in vortex for 1 minute intercalated for 2 minutes on ice. The tubes were kept at room temperature to let the glass beads to precipitate. A second Trizol extraction was conducted according to the manufacturer’s instructions. At the end, the RNA was diluted in DEPC-treated water and stored in the freezer in separate aliquots. cDNA synthesis was done with the RevertAid H minus First Strand cDNA synthesis kit (Thermo Scientific) from 5 μg of total RNA. Real time PCR amplifications were made from diluted templates (1:10) with 600 nM target primers, 300 nM of internal control primers (*GPDH*1—Glyceraldehyde-3-phosphate dehydrogenase), and 1X Power SYBR Green master mix (Life Technologies). Quantification of the transcript levels was performed using the *ΔΔ*^*CT*^ method normalizing against *GPDH*1, as previously described [58]. Analysis of variance was performed by Tukey′s multiple comparison test using GraphPad Prism 5.0 software, and *p* values lower than 0.05 were considered statistically significant.

## Results

### *C*. *neoformans* permease genes are regulated by temperature

**Fig 1:**
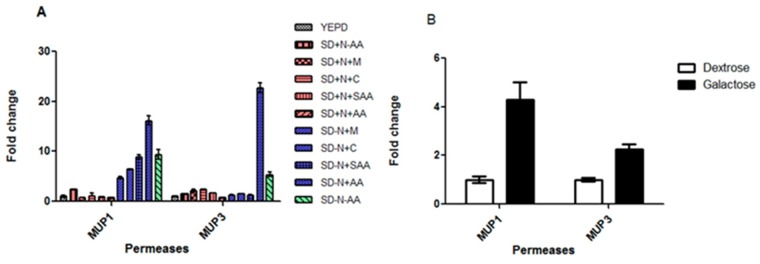
qPCR of the *MUP* genes in response to nitrogen sources (A) and carbon sources (B), dextrose versus galactose. In (A) Colors groups represent different nutritional conditions: white = rich medium (YEPD), red = synthetic medium (SD) supplemented with preferred nitrogen source (with ammonium sulfate + selected amino acids), blue = SD (without ammonium sulfate) plus selected amino acids and green = nitrogen starvation. The pattern of the bars represents different medium composition, according to the legend. SD = synthetic dextrose; − or +N = ammonium sulfate omitted or added; − or +C = cysteine omitted or added; − or +M = methionine omitted or added; − or + SAA = cysteine and methionine omitted or added; − or + AA = tryptophan, hystidine and methionine omitted or added, respectively. Statistically significant differences at *p* value < 0.05.

Our previous study demonstrated that *C*. *neoformans* has ten permeases, whereas *S*. *cerevisiae* has twenty-four, considering the APC-superfamily, which predominates in fungi [39,40]. The genomic arrangement of permeases is similar among the Basidiomycetes analyzed, that is, eight global permeases (Aap1 to 8) and one to two sulfur amino acid permeases (Mup1 and Mup3) with a high and low affinity for methionine and cysteine. Our previous data also showed that permeases genes *AAP*3 and *AAP*7 are not expressed in YEPD, SD added with ammonium sulfate and SD added with amino acids; *AAP*6 displayed no transcriptional change according to nitrogen source (ammonium sulfate or amino acids) and *AAP*8 showed increased expression in the presence of amino acids only; permeases *AAP*2, 4 and 5 had the highest transcriptional induction in the presence of amino acids (24, 49 and 111-fold induction respectively). Also, *AAP*2 and 5 are under NCR control and *AAP*4 is not [27]. However, the two amino acid permeases that resemble *S*. *cerevisiae* Mup1 and Mup3 remain uncharacterized. Therefore, we started to analyze these permease genes; as a first step towards this goal, we determined the transcriptional profile of *MUP*1 and *MUP*3 (Fig 1A) according to the nutritional status by qPCR. As shown in Fig 1A, both genes are under NCR regulation (red bars in Fig 1A). However, once the NCR is alleviated (blue bars in Fig 1A), the highest degree of induction can be achieved by the addition of a pool of amino acids SD-N+AA (histidine, tryptophan and methionine). In addition, *MUP*1 is induced by sulfur amino acids (SAA), either in combination (SD-N +SAA) or separately (SD-N+C or SD-N+M), whereas *MUP*3 is not. Since we knew Carbon Catabolite Repression (CCR) also plays a role in *AAP*s permease regulation [27], we tested the transcriptional induction under preferred and non-preferred carbon sources (glucose and galactose, respectively). Fig 1B shows that, similarly to the permeases encoded by *AAP* genes, *MUP*1 and *MUP*3 also undergo transcriptional induction in the presence of a non-preferred carbon source.

**Fig 2:**
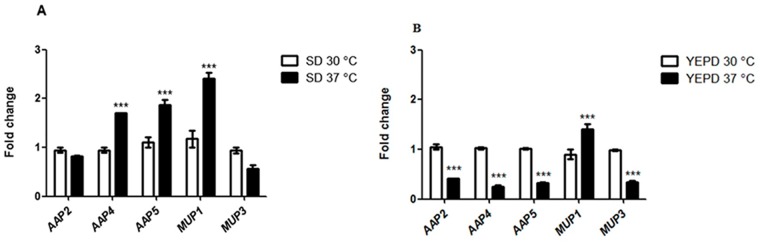
Effect of growth temperature (30°C and 37°C) on expression pattern of permease genes. Growth conditions are synthetic medium (A) and rich medium (B). SD = synthetic dextrose added with ammonium sulfate (without amino acids). Stars represent statistically significant differences at *p* value < 0.05.

Subsequently, we investigated if temperature would modulate the transcription of the permeases. First, pemeases *AAP*6 and *AAP*8 were not regulated by temperature. Fig 2A shows the transcriptional profile of *AAP*2, *AAP*4, *AAP*5, *MUP*1 and *MUP*3 genes at 30°C and 37°C in synthetic medium supplemented with ammonium sulfate (no amino acid), and Fig 2B shows the same permeases in rich medium at 30°C and 37°C. *AAP*4, *AAP*5 and *MUP*1 are induced by temperature shift (30°C to 37°C) in synthetic dextrose as shown in Fig 2A. Conversely, in rich medium (YEPD), which is known to promote transcriptional repression relative to synthetic medium, a higher temperature leads to even further transcriptional inhibition, as shown in Fig 2B (60 to 78% inhibition). The fact that temperature imposes transcriptional control over permease genes may connect amino acid transport and pathogenesis, since high temperature growth is an essential feature of host invasion [59–62].

### Permease substrate specificity and high-temperature growth

In order to gain insights into amino acid uptake mechanism, permease specificity and its implications in high temperature growth and virulence, we chose five permease genes to be deleted from the *C*. *neoformans* genome and conducted a phenotypic characterization of the mutants: *AAP*2 (CNAG_07902), *AAP*4 (CNAG_00597), *AAP*5 (CNAG_07367), *MUP*1 (CNAG_07693), and *MUP*3 (CNAG_03955). This choice was based on several factors: (i) enhanced expression in synthetic medium; (ii) increased transcription in the presence of amino acids; (iii) all of the previously listed genes are subject to NCR, except for *AAP*4, [27]; and (iv) transcriptional response to high-temperature growth, as shown in Fig 2. Furthermore, *AAP*4 and 5 seem to encode very similar proteins (89% similarity at the amino acid level), which is suggestive of protein redundancy and, therefore, they may have a central role in amino acid transport [27].

Moreover, an argument in favor of dissecting the role of *MUP*1 and *MUP*3 is the fact that they are the only genes in the *C*. *neoformans* genome encoding permeases that seem to transport specific amino acids (namely, the sulfur amino acids cysteine and methionine).

Hence, we deleted all five genes individually from the start to stop codon by substituting the coding sequence with a dominant resistance marker, conferring resistance to G418 (*Neo*^R^) or hygromycin (*Hph*^R^) (S1 Fig). In addition to that, two double mutants were constructed due to the possibility of gene redundancy: *aap*4Δ::*Hph*^R^/*aap*5Δ::*Neo*^R^ and *mup*1Δ::*Neo*^R^/*mup*3Δ::*Hph*^R^.

**Fig 3:**
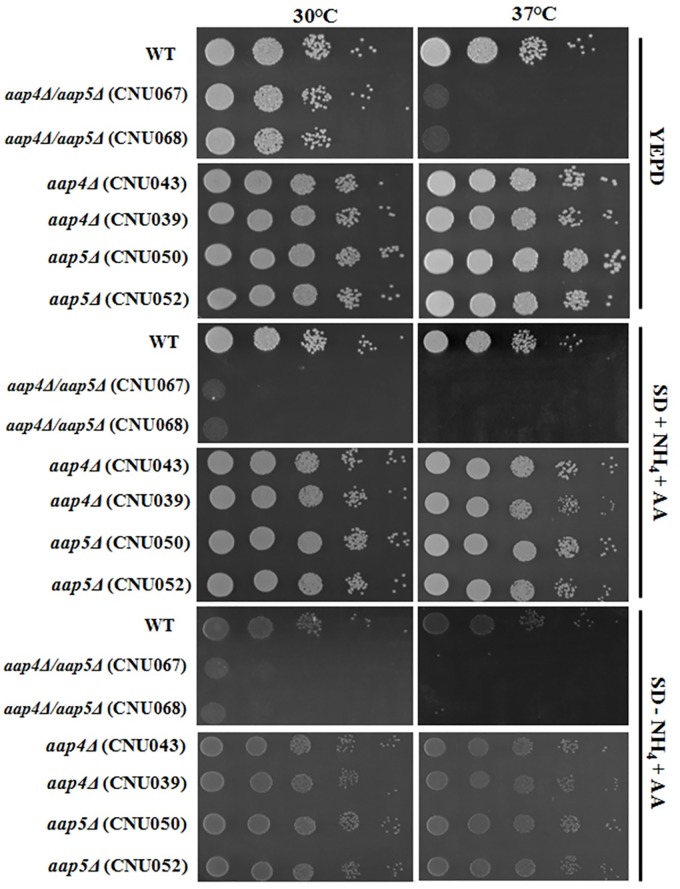
Growth phenotype of wild type, single (*aap*4Δ and *aap*5Δ) and the double mutant *aap*4Δ/*aap*5Δ (two independent transformants of each) in rich medium YEPD, synthetic dextrose supplemented with ammonium sulfate and amino acids and synthetic dextrose supplemented with amino acids only at 30 and 37°C. Serial dilutions represent 10^4^, 10^3^, 10^2^, 10^1^ and 1 cell.

The initial mutant characterization indicated that the individual deletion of *Aap*2, *Mup*1, *Mup*3 and also the double deletion (*mup*1Δ::*Neo*^R^/*mup*3Δ::*Hph*^R^) did not change the growth rate of these strains compared to the wild type, neither in rich nor in synthetic medium supplemented with ammonium sulfate and amino acids or amino acids as sole nitrogen source at 30°C and 37°C, indicating that their general fitness is not disturbed by the deletion of individual permeases (data not shown). Also, no difference was observed for the single mutants *aap*4Δ and *aap*5Δ strains in all growth conditions tested (Fig 3). In rich medium (YEPD) the double mutant *aap*4Δ::*Hph*^R^/*aap*5Δ::*Neo*^R^ grew to the same rate as wild type at 30°C, but showed significant decrease in growth at 37°C. The double mutant also grew poorly in synthetic dextrose supplemented with ammonium sulfate and amino acids at 30°C and no growth was observed at 37°C (Fig 3). The use of amino acids as sole nitrogen source impaired growth of the double mutant at both temperatures 30 and 37°C (Fig 3). This result suggests that Aap4 and Aap5 permeases are important during high temperature growth.

**Fig 4:**
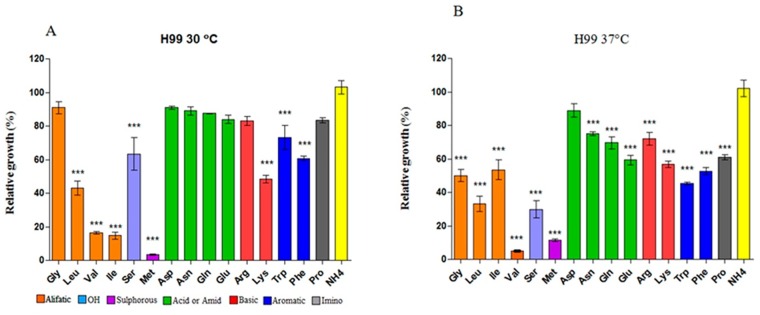
Amino acid uptake by *C*. *neoformans* wild type. Percentage growth of H99 on 19 amino acids as sole nitrogen sources relative to ammonium sulfate at 30°C (A) and 37°C (B). Colored bars indicate biochemical groups of amino acids: aliphatic (orange), hydroxilated (purple), sulfur (pink), acid/amide (green), basic (red), aromatic (blue), imino (grey). The yellow bar represents growth in the preferred nitrogen source, ammonium sulfate, which represents 100% growth. Statistically significant difference denoted by a * (*p* < 0.05)

Even though the sequence similarity data indicated that *AAP*2, *AAP*4, *AAP*5 encode global permeases and *MUP*1 and *MUP*3 encode high- and low-affinity methionine permeases, we used the mutants to answer the following question: are these permeases in fact responsible for general or specific amino acid transport? In order to address this question, we tested to what extent individual amino acid, as the sole nitrogen source, could support the growth of the wild type and then compared that to the individual and double mutants. First, we determined the growth rate of the wild type H99 grown on 10 mM of each amino acid as the sole nitrogen source at 30°C and 37°C. We concluded that alanine, cysteine, threonine and histidine did not support growth at either 30°C or 37°C. These amino acids support less than 2% of growth when compared to the preferred nitrogen source (ammonium sulfate) and, therefore, we considered that *C*. *neoformans* does not use them as nitrogen sources. Valine, leucine, isoleucine and methionine were considered poor nitrogen sources, since growth was detected, albeit at low rate (between 4 and 40% relative to ammonium sulfate). All other amino acids tested were considered good nitrogen sources, conferring at least 50% growth relative to ammonium sulfate, even though some showed statistically significant differences when compared to ammonium sulfate (serine, lysine, tryptophan and phenylalanine). Seven amino acids promoted growth at the same level as did ammonium sulfate (no statistical significance), as shown in Fig 4A. These results are in agreement with the literature, except for alanine and glutamic acid, which were considered preferred and non-preferred nitrogen sources, respectively in another study [63]. Moreover, we did not include tyrosine in our test, since this amino acid is only soluble at pH 2.0, which renders growth unviable for *C*. *neoformans*. Therefore, fifteen amino acids could be tested as sole nitrogen sources.

Taking into account the biochemical nature of the amino acids side chains, we could readily identify that amides (glutamine and asparagine) and acidic amino acids (aspartate and glutamic acid) are the best nitrogen sources among the nineteen amino acids, since they all promote very similar growth rates as does the preferred nitrogen source, ammonium sulfate (green bars in Fig 4A). Other amino acids (glycine, arginine and proline) also promote growth rates similar to those achieved with the preferred nitrogen source, but statistically significant differences were identified.

The same experiment at 37°C showed that, in general, the growth rate on amino acids was lower when compared to 30°C. Fig 4B shows that only on aspartic acid the growth rate was not statistically different than that observed on ammonium sulfate. All other amino acids, as sole nitrogen sources, differ significantly from ammonium sulfate. One possible explanation for this result is that, at higher temperatures, permease conformation is less stable, leading to less efficient amino acid transport and, therefore, to lower growth rates. Our previous data showed that a tryptophan auxotroph can be partially remedied when NCR is alleviated by the use of proline as a nitrogen source, especially at lower temperatures [27] and the same thing holds true for a threonine auxotroph previously described [28].

Once the growth rate on single amino acids was found for the wild type (H99), we submitted five single mutants (*aap*2Δ::*Neo*^R^, *aap*4Δ::*Neo*^R^, *aap*5Δ::*Neo*^R^, *mup*1Δ::*Neo*^R^, *mup*3Δ::*Hph*^R^) and the two double mutants (*aap*4Δ::*Hph*^R^/*aap*5Δ::*Neo*^R^ and *mup*1Δ::*Neo*^R^/*mup*3Δ::*Hph*^R^) to the same experiment in order to learn if any of these mutations would lead to differences in growth compared to the wild type, where single amino acids were used as the sole nitrogen source. Statistically significant differences between the wild type and mutant correlate a lower growth with the lack of permease function, suggesting lower amino acid transport.

### The Aap2 is a global amino acid permease

Growth rate experiments performed with an *aap2* mutant and wild type at 30°C showed that the deletion of permease *AAP*2 led to a significant lower growth on arginine, lysine and isoleucine as sole nitrogen sources (14%, 40% and 28% relative to the wild type, respectively). All other amino acids had assimilation profiles very similar to H99 (Table 1).

**Table 1:** Growth rate of *C*. *neoformans* wt and mutants.

Aminoacid group	Nitrogen source	*aap*2Δ	*aap*4Δ	*aap*5Δ	*aap*4Δ/ *aap*5Δ	*mup*1Δ	*mup*3Δ	*mup*1Δ/ *mup*3Δ
Aliphatic	Alanine	-	-	-	-	-	-	-
	Glycine	NS	58% (*)	60% (*)	NS	NS	NS	NS
	Isoleucine	28% (***)	27% (***)	36% (***)	22% (***)	NS	NS	NS
	Leucine	NS	NS	NS	6% (***)	NS	NS	NS
	Valine	NS	57% (**)	NS	51% (**)	NS	NS	43% (**)
Hydroxylated	Serine	NS	44% (**)	NS	10% (***)	NS	NS	58% (*)
	Threonine	-	-	-	-	-	-	-
Sulphur	Cysteine	-	-	-	-	-	-	-
	Methionine	NS	11% (**)	NS	NS	NS	NS	30% (*)
Acidic/Amide	Asparagine	NS	NS	NS	51% (**)	NS	NS	NS
	Aspartic acid	NS	13% (***)	NS	7% (***)	NS	NS	NS
	Glutamic acid	NS	NS	NS	4% (***)	NS	NS	NS
	Glutamine	NS	NS	NS	46% (**)	NS	NS	NS
Basic5	Arginine	14% (***)	NS	NS	20% (***)	NS	NS	NS
	Histidine	-	-	-	-	-	-	-
	Lysine	40% (***)	36% (***)	NS	11% (***)	NS	NS	NS
Aromatic	Phenylalanine	NS	49% (***)	NS	42% (***)	NS	NS	NS
	Tryptophan	NS	NS	NS	58% (**)	69% (*)	34% (***)	40% (***)
	Tyrosine	-	-	-	-	-	-	-
Imino	Proline	NS	NS	59% (**)	32% (***)	83% (*)	NS	81% (**)

Growth percentage of each permease mutant (single and double mutant) relative to that of the wild type (H99) on single amino acid as the sole nitrogen source at 30°C. Non-significant differences and no growth are denoted as “NS” and “-”, respectively; *p* value < 0.05 (*), 0.01 (**), 0.0001 (***).

The same experiment was conducted at 37°C and a more severe effect could be observed (Table 2). Growth was moderately reduced on aliphatic (glycine), aromatic (phenylalanine and tryptophan), sulfur (methionine) and acidic/amide (aspartic acid, glutamic acid, asparagine and glutamine) amino acids used as the sole nitrogen source.

**Table 2:** Growth rate of *C*. *neoformans* wt and mutants.

Aminoacid group	Nitrogen source	*aap*2Δ	*aap*4Δ	*aap*5Δ	*aap*4Δ/ *aap*5Δ	*mup*1Δ	*mup*3Δ	*mup*1Δ/ *mup*3Δ
Aliphatic	Alanine	-	-	-	-	-	-	-
	Glycine	59% (***)	NS	NS	0% (***)	83% (*)	75% (**)	83% (*)
	Isoleucine	NS	NS	NS	20% (***)	NS	NS	55% (**)
	Leucine	NS	NS	NS	6% (***)	NS	NS	NS
	Valine	NS	NS	9% (***)	4% (***)	28% (***)	8% (**)	17% (***)
Hydroxylated	Serine	NS	NS	NS	12% (***)	NS	NS	NS
	Threonine	-	-	-	-	-	-	-
Sulphur	Cysteine	-	-	-	-	-	-	-
	Methionine	64% (**)	NS	44% (***)	4% (***)	44% (***)	20% (***)	10% (***)
Acidic/amide	Asparagine	61% (***)	NS	NS	0.4% (***)	96% (*)	81% (**)	72% (***)
	Aspartic acid	30% (***)	70% (**)	59% (***)	1% (***)	72% (**)	74% (**)	60% (***)
	Glutamic acid	58% (**)	73% (**)	NS	0.7% (***)	NS	NS	NS
	Glutamine	77% (*)	NS	NS	0% (***)	NS	NS	79% (*)
Basic	Arginine	19% (***)	72% (**)	NS	3% (***)	NS	75% (**)	61% (***)
	Histidine	-	-	-	-	-	-	-
	Lysine	72% (**)	NS	NS	4% (***)	NS	81% (*)	NS
Aromatic	Phenylalanine	48% (***)	NS	73% (**)	0% (***)	NS	78% (*)	79% (*)
	Tryptophan	67% (***)	NS	NS	0% (***)	NS	82% (**)	85% (**)
	Tyrosine	-	-	-	-	-	-	-
Imino	Proline	NS	NS	76% (**)	58% (***)	80% (*)	NS	73% (**)

Growth percentage of each permease mutant (single and double mutant) relative to that of the wild type (H99) on single amino acid as the sole nitrogen source at 37°C. Non-significant differences and no growth are denoted as “NS” and “-”, respectively; *p* value < 0.05 (*), 0.01 (**) and 0.001 (***).

These results suggest that permease Aap2 is a global amino acid transporter especially at higher temperatures. Since we did not detected Aap2 transcriptional induction at 37°C (Fig 2A), we would think that this effect is due to the protein conformation rather than any effect of the temperature on gene regulation. In this regard, one can infer that amino acid transport is an important nutritional mechanism that plays a role at higher temperatures in order to guarantee proper nutrient supply. Aap2 may be well adapted to this condition, that is, it may be still stable and efficient at 37°C, allowing amino acid transport to occur in a hostile temperature. In agreement with our observation, recently, Do *et*. *al*. (2016) reported that permeases Aap2 and Aap3 are induced by the use of lysine as the sole nitrogen source [64].

### Mup1 and Mup3 act as global amino acid transporters

Our bioinformatics studies indicated that *MUP*1 and *MUP*3 may encode high- and low-affinity methionine permeases, respectively [65,66]. If that is the case, deletion of each gene separately or in combination should affect the uptake of sulfur amino acids. Cysteine was not considered as a nitrogen source that supports growth; therefore, it was not possible to identify its utilization profile by wild type and mutants. Even though methionine was considered a poor nitrogen source, it still supported the growth of H99 and, hence, was considered in this analysis. Table 1 shows the relative growth of *mup*1Δ::*Neo*^R^, *mup*3Δ::*Hph*^R^ single and double mutant at 30°C on amino acids. The results showed that the growth on single amino acid as the sole nitrogen source was not affected by the mutations, except when using (i) tryptophan (reduction in the growth of all three mutants); (ii) proline, (growth reduction for *mup1* and the double mutant); (iii) methionine, (iv) valine and (v) serine, all of which supported low growth in the double mutant strain. At 37°C, the two single and the double mutants had a more prominent effect regarding growth efficiency. Statistically significant growth differences were observed when single amino acids were used as the sole nitrogen sources (Table 2). In spite of the gene deletion effect being broad, that is, out of 19 amino acids tested, eleven were poorly assimilated in the double mutant relative to the wild type, growth reduction was moderate (Table 2), except for valine and methionine, for which growth was reduced 83 and 90%, respectively. These results strongly suggest that Mup1 and Mup3 are not methionine and cysteine high- and low-affinity permeases, but they act as global permeases, and are required for growth at high temperatures as well.

### *AAP4* and *AAP5* encode global permeases essential to high temperature growth and oxidative stress

The single deletion of *AAP*4 and *AAP*5 led to moderate growth reduction in single amino acid as sole nitrogen source at both, 30° and 37°C (Tables 1 and 2, respectively). The double deletion of the permease-coding genes *AAP*4 and *AAP*5 resulted in the highest impact on growth. Out of the fifteen amino acids tested as the sole nitrogen source at 30°C, thirteen supported low growth in the double mutant relative to wild type, that is, only glycine and methionine promoted growth rates similar to wild type (Table 1). However, at 37°C, the deletion of both permeases drastically reduced the growth on all of the single amino acids tested (Table 2). These results show that Aap4 and Aap5 are important global amino acid permeases, both of which are highly redundant and essential for amino acid uptake, especially at 37°C.

**Fig 5:**
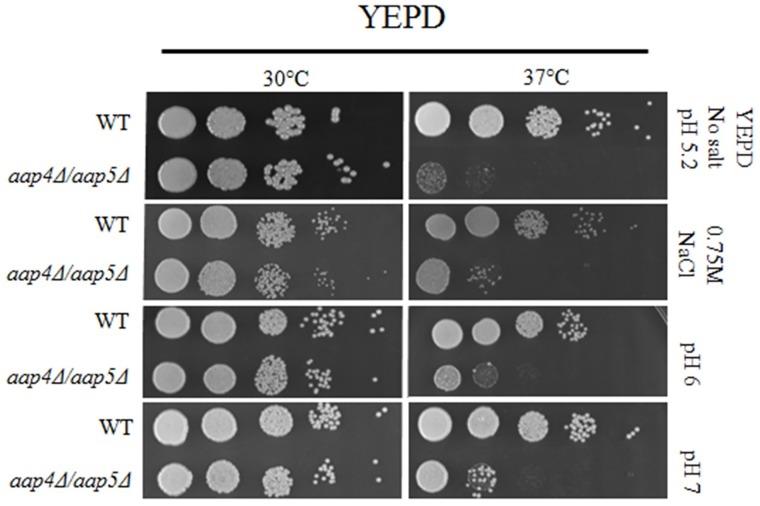
Growth pattern of H99 and double mutant (*aap*4Δ/*aap*5Δ) in YEPD at pH 5.2 without supplements or supplemented with NaCl (0.75 M), in YEPD at pHs 6 and 7 (without salt supplements). Serial dilutions represent 10^4^, 10^3^, 10^2^, 10^1^ and 1 cell. *aap*4Δ and *aap*5Δ single mutants under the same conditions as reference are presented in S2 Fig.

Interestingly, the growth defect observed for the double mutant *aap*4Δ::*Hph*^R^/*aap*5Δ::*Neo*^R^ in rich medium at 37°C was remedied in high salt (0.75 M NaCl), and alkaline conditions (pH 6 and 7) as shown in Fig 5. The growth rate of the single mutants (*aap*4Δ and *aap*5Δ) relative to the wild type was not affected by high salt and alkaline pH (S2 Fig). One interpretation for this result is that the members of the APC-superfamily are proton-driven permeases, which may benefit from excess ions, leading to a more efficient amino acid transport, compensating for the deletion of *AAP*4 and *AAP*5.

**Fig 6:**
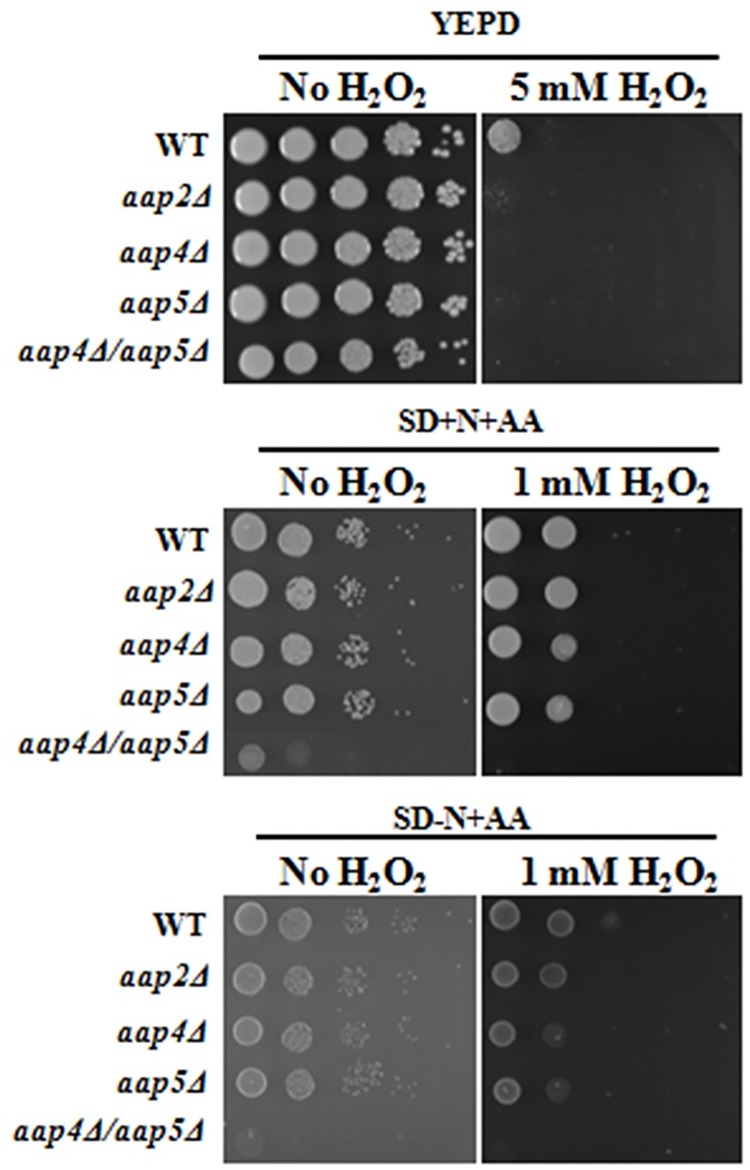
Growth rate in wild type and mutants under oxidative stress. Serial dilution (10^4^, 10^3^, 10^2^, 10^1^ and 1 cell) were spotted on YEPD not supplemented and supplemented with 5 mM H_2_O_2_ (A) and SD supplemented with ammonium sulfate and SD supplemented with amino acids only with 1 mM H_2_O_2_ (B). Growth was carried out at 30°C up to 72 hours.

All mutants were also tested for their ability to resist oxidative stress compared to the wild type. Regarding *mup* mutants (singles and double mutants) no difference was detected relative to wild type in all conditions tested (rich and SD media). However, as shown in Fig 6, all *AAP* mutants (single and double) are sensitive to 5 mM H_2_O_2_ in rich medium (YEPD). Bellow this concentration, all strains behaved as wild type. In SD supplemented with ammonium sulfate and amino acids and SD supplemented with amino acids only plus 1 mM H_2_O_2_, the single mutants were only slightly more sensitive to oxidative stress than wild type. The double mutant *aap*4Δ/*aap*5Δ was highly sensitive to oxidative stress in SD plus ammonium sulfate and amino acids; as expected this double mutant did not grow on amino acids as sole nitrogen source (Fig 6). This result suggests that amino acid permeases are important, not only for thermal, but also for oxidative stress resistance and permeases Aap4 and 5 may be important to mediate the amino acid uptake during these stress conditions.

Taken together, these experiments unraveled important points about amino acid uptake, namely: (i) there are at least five global permeases that transport amino acids; (ii) among these, three are minor (Aap2, Mup1 and Mup3) and two (Aap4 and Aap5) are major permeases; (iii) high temperature growth requires amino acid permeases; (iv) the lack of Aap4 and Aap5 can be remedied by high salt and alkaline conditions (high pH); (v) amino acid permeases are required under oxidative stress conditions as well.

Consequently, we can conclude that Aap4 and Aap5 act as redundant and dominant permeases in *C*. *neoformans*. Amino acid permeases are required during heat and oxidative stress, two major conditions encountered during host invasion. This conclusion prompted us to check the effect of permease deletion on virulence *in vitro* and *in vivo*.

### The role of amino acid permeases Aap4, Aap5, Mup1 and Mup3 in capsule production

**Fig 7:**
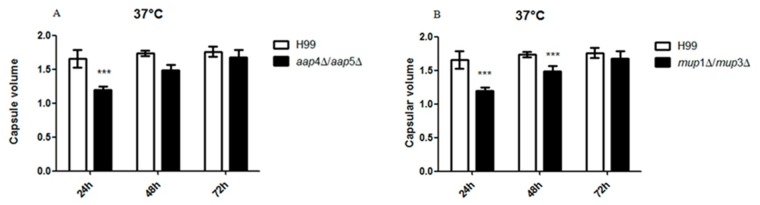
Capsule production in the double mutant *aap*4Δ/*aap*5Δ (A) and *mup*1Δ/*mup*3Δ (B) at 24, 48 and 72 hours post inoculation at 37°C. Asterisks represent significant differences compared to wild type H99 at a *p* value < 0.05.

Once the role of the permeases in amino acid uptake was established, we verified whether they are associated with the main virulence traits and other stress resistance in *C*. *neoformans*. We examined all single mutants and double mutants for their resistance to plasma membrane and cell wall stressor (Congo red and SDS), the ability to produce melanine, urease and phospholipase. No impact on these traits was detected in any one of the mutants (data not shown). In regard to capsule synthesis, one of the main virulence factors in *C*. *neoformans*, there was a significant delay in its production, especially at 24 hours in the case of the two double mutants (*aap*4Δ::*Hph*^R^/*aap*5Δ::*Neo*^R^ and *mup*1Δ::*Neo*^R^/*mup*3Δ::*Hph*^R^) when compared to the wild type (H99) at 37°C (Fig 7A and 7B). No difference was observed for the double mutants and wild type at 30°C.

### Aap4 and Aap5 are required for virulence in an animal model

**Fig 8:**
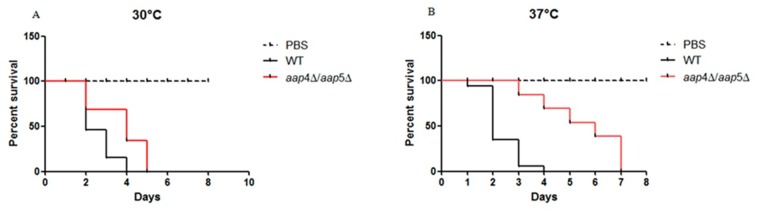
Virulence in *G*. *mellonella*. Larvae were inoculated with either the wild type (H99) or the double mutant *aap*4Δ/*aap*5Δ and incubated at 30 (A) and 37°C (B). Significant differences were considered at *p* values <0.05 for the comparisons between wild type and mutants.

Since our results showed that amino acid permeases Aap4 and Aap5 are required for an important virulence factor (capsule) and stress resistance we were motivated to test all the singles (*aap*2Δ, *aap*4Δ, *aap*5Δ, *mup*1Δ and *mup*3Δ) and double mutants (*aap*4Δ::*Hph*^R^/*aap*5Δ::*Neo*^R^ and *mup*1Δ::*Neo*^R^/*mup*3Δ::*Hph*^R^) in the invertebrate animal model *Galleria mellonella*. Larvae inoculation with wild type and mutants were incubated at 30°C and 37°C. At both of these temperatures, the double mutant *aap*4Δ::*Hph*^R^/*aap*5Δ::*Neo*^R^ was hypovirulent in the *G*. *mellonella* animal model. As shown in Fig 8A (30°C), by day four all larvae inoculated with H99 had died and the ones inoculated with the double mutant died by day five. Larvae inoculated with the double mutant and kept at 37°C (Fig 8B) died by day seven following inoculation. For the other mutants, no difference in comparison to the wild type was found, suggesting that they are not essential for virulence (data not shown).

**Fig 9:**
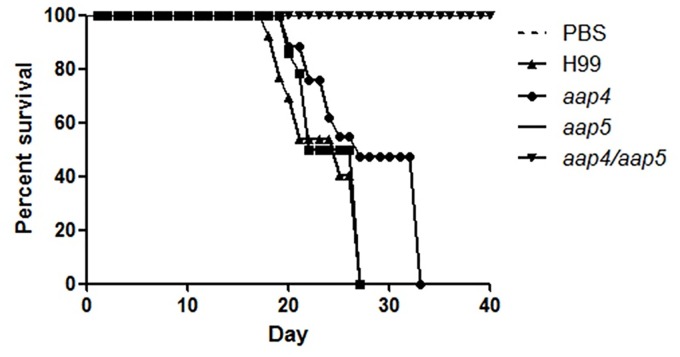
Virulence test in animal model. Balb/C mice (10/group) were infected by nasal inhalation method with wild type (H99), *aap*4Δ, *aap*5Δ and the double mutant *aap*4Δ/*aap*5Δ. Survival was followed during the course of the infection. *p* values were <0.05 for the comparisons between wild type and mutants.

This result motivated us to further test the virulence of the double mutant strain (*aap*4Δ::*Hph*^R^/*aap*5Δ::*Neo*^R^) in a vertebrate animal model. The experiment was carried out for H99, two single mutants (*aap*4Δ::*Neo*^R^ and *aap*5Δ::*Neo*^R^) and one double mutant (*aap*4Δ::*Hph*^R^/*aap*5Δ::*Neo*^R^). The results showed that the mice inoculated with the single mutants and the wild type H99 lost viability between days 28 and 32 post-inoculation. These strains were, therefore, considered virulent. However, 100% of the mice inoculated with the double mutant and PBS survived to the end of the experiment (40 days), indicating that both permeases, Aap4 and Aap5, are essential for virulence (Fig 9) and their gene knockouts rendered *C*. *neoformans* avirulent, which suggests, once more, that they play redundant and important roles in this yeast.

Also, colony counts in the organs (lungs, liver brain and spleen) extracted from 5 mice inoculated with the double mutant that survived 40 days following inoculation showed viable yeasts in the lungs and liver. Several of the colonies recovered were tested for selectable markers and all of them were confirmed to be from the double mutant.

**Fig 10:**
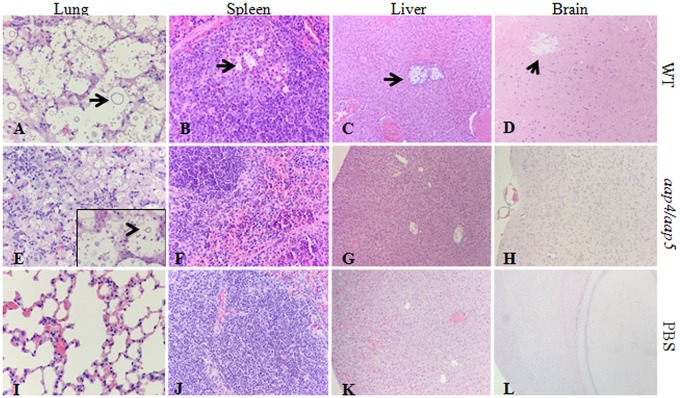
Effects of Amino acid permeases double mutant on *in vivo* virulence. Representative photomicrographs of lungs, spleen, liver and brain from mice infected with H99, double mutant *aap*4Δ/*aap*5Δ and negative control (PBS), H&E stain. Histopathology analysis showed infection restricted to the lungs and increased inflammatory cell infiltration in double-mutant infected mice. Black arrows mark *C*. *neoformans* cells. In detail, arrowhead indicates budding yeast cell. Magnification: A, B, E, F, I, J x100; E, (in detail) x400; C, D, G, H, K, L x40.

The histopathological analysis of lungs, spleen, liver and brain showed that the wild type and the single mutants caused very similar effects in these tissues. As shown in Fig 10, extensive proliferation of yeast with titan cells in the alveolar space and a very limited inflammatory response, resulting in subsequent alveolar granulomas, can be observed in the tissues of the mice inoculated with H99. In addition, yeast cells were observed in spleen, liver and brain tissue (arrows in panels A, B, C and D). However, in mice inoculated with the double mutant, budding yeasts were identified only in the lungs (arrowhead in panel E), where the tissue was observed to be much more infiltrated with prominent macrophage response, some multinucleated giant cells, lymphocytes and plasma cells. Indeed, in these cases, the infection was restricted to lungs, while other organs were much more similar to the negative control (inoculated with PBS only).

Taken together, these results showed that amino acid uptake mediated by permeases Aap4 and Aap5 is an essential mechanism, required for resisting thermal and oxidative stress, which helps *C*. *neoformans* to survive in and colonize the animal host.

### Amino acid permeases and antifungal sensitivity

Among the five permeases studied in this research, the deletion of *AAP*4 and *AAP*5 caused hypersensitivity to fluconazole. The single and double mutants were six fold (0.25 μg/mL) and twelvefold (0.125 μg/mL), respectively, as sensitive to fluconazole as the wild type and the strain deleted for Aap2 (1.5 μg/mL), as shown in Table 3. All strains (wild type, single mutant and double mutant) had the same sensitivity to amphotericin B. This result suggests that amino acid uptake may specifically play an important role during fluconazole response; however, the mechanistic bases of the response are not yet known.

**Table 3:** Minimum inhibitory concentration determined by Etest in RPMI medium at 30°C.

Strain	Fluconazol	
	μg/mL	% inhibition
H99	48	95
*aap2*Δ	64	80
*aap4*Δ	12	100
*aap5*Δ	12	100
*aap4*Δ/*aap5*Δ	8	100

Readings were taken 72 hours following incubation.

Literature reports pointed out that eugenol, an aromatic plant metabolite extracted specially from cloves and other plants, has a potent antifungal activity due to perturbations to the uptake of aromatic and branched-chain amino acids. Moreover, it has been shown that *S*. *cerevisiae* Tat2 and Gap1 permeases are both targets of eugenol [49]. According to the authors, the explanation for this phenomenon lies on the similar structural conformation of eugenol and phenylalanine. In this study, we determined the MIC of eugenol in the wild type, single and double mutants. Table 4 shows that *C*. *neoformans* wild type strain H99 is sensitive to eugenol (0.4 μg/μL), and individual permease inactivation (*aap*2, 4 and 5) leads to a fourfold increase in resistance (1.6 μg/μL) to eugenol when compared to the wild type, whereas the double mutant *aap*4Δ/*aap*5Δ is 7.75-fold (3.1 μg/μL) more resistant than H99.

**Table 4:** Eugenol minimum inhibitory concentration.

Strain	μg/μL Eugenol
H99	0.4 (90 ± 9)
*aap2*Δ	1.6 (90 ± 6)
*aap4*Δ	1.6 (94 ± 1)
*aap5*Δ	1.6 (93 ± 2)
*aap4*Δ/*aap5*Δ	3.1 (95 ± 1)

Numbers in parenthesis show the average growth inhibition and standard deviation at the MIC of eugenol (μg/μL). The experiments were conducted as biological triplicates.

Taken together, these results show that amino acid permeases are important for pathogenesis, required for *in vivo* survival, and also that they are the target of inhibitors, which underlines their therapeutic potential.

## Discussion

Opportunistic yeasts often occupy very different environments. *C*. *neoformans* colonizes decaying wood, pigeon guano and animal hosts, all of which may offer completely different types of nutrients, sometimes at limited amounts, such as of carbon, sulfur, iron, phosphate and nitrogen [6,9,11]. A series of articles have shown that amino acid biosynthetic pathways are very important during host invasion in *C*. *neoformans* and, therefore, have a potential as drug targets [28–34]. More recently, the threonine and tryptophan biosynthetic pathways have been shown to be essential for *C*. *neoformans* survival *in vitro* and the low efficiency in amino acid uptake arose as the reason for the auxotroph’s lack of complementation [27,28].

Uptake is dependent upon amino acid permeases, which have been described in *C*. *neoformans* by our group but not dissected in detail. Previously, our study had showed that *C*. *neoformans* and other basidiomycetes have a very different set of amino acid permeases when compared to ascomycetes. The more scientists explore the biology of basidiomycetes, the more we can detect differences between them and the ascomycetes, which are by far more investigated and, therefore, serve as a frame of reference. The permease genes are a good example of diversity between these two phyla in the fungal domain. Another example is pH response in *C*. *neoformans*, in which the outcome of the signaling pathway is the same, even though the players are different [26].

Our previous data [27] and the extended bioinformatics investigation conducted for this study showed that the permease gene organization in *C*. *neoformans* is closer to *C*. *gattii*, *U*. *maydis* and *C*. *cinerea* than to *S*. *cerevisiae*, *C*. *albicans* and *Aspergillus* spp. According to our sequence similarity analysis, six permeases are global and two are methionine/cysteine permeases. On the other hand, in *S*. *cerevisiae* there are twenty-four APC-like permeases, with most of them transporting few amino acids and Gap1 being the main high capacity and global permease [37–40,67]. The *C*. *albicans* genome encodes twenty-seven putative permeases and six *GAP*1 homologues and one of them, *GAP*2, act as sensor and transporter [43].

In this study, we extended our transcriptional analysis and showed that permeases controlled by the nitrogen quality (NCR) are also under the influence of temperature, an important virulence factor in *C*. *neoformans*. Further details on how the permease genes are regulated and what are the players that control their expression will be published elsewhere. In this paper, deletion and phenotypic analysis of these five genes, individually or in combination, correlate them to amino acid uptake, stress resistance, virulence and antifungal resistance. Our main findings showed that (i) all of the permeases studied are global (Tables 1 and 2); (ii) amino acid permeases are very important during high temperature growth and oxidative stress response (Figs 2, 3 and 6, Tables 1 and 2); (iii) Aap4 and Aap5 encode the two major redundant permeases with broad substrate spectrum, high transport efficiency and are essential during the response to stressors (Tables 1 and 2, Figs 3 and 6); (iv) the growth deficiency at 37°C caused by the lack of Aap4 and Aap5 can be remedied by Na, and alkaline conditions (high pH) (Fig 5), which is consistent with proton-driven type of permeases of the APC-superfamily; (v) amino acid permeases Aap4 and Aap5 are important for correct timing of capsule synthesis, and virulence *in vivo* in invertebrate and vertebrate animal models (Figs 7, 8, 9 and 10); and finally (vi) Aap4 and Aap5 permeases are required for fluconazole resistance (Table 3) and are useful as the target of inhibitors, such as the plant secondary metabolite eugenol (Table 4), an antifungal agent that targets amino acid permeases in *S*. *cerevisiae* [49].

Aap4 and Aap5 are necessary during thermal and oxidative stress, consistent with the role of both physiological responses in host invasion and virulence. Other nutrient transporters, such as glucose, ammonium and iron transporters, have been studied in pathogenic fungi and shown to affect fitness, *in vitro* survival and to attenuate virulence in some cases [8,68–70]. A previous report related the same effects for the glucose transporter in *C*. *neoformans* [68]. To our knowledge, this is the first time that amino acid transport is reported as important for *in vivo* virulence in an opportunistic pathogen that affects humans. In *C*. *albicans*, the amino acid sensor protein Csy1 induces permease gene transcription and amino acid uptake, which is an important mechanism for hyphal development; however, it is not known whether this morphogenetic defect influences virulence [45].

Recently, the closest direct correlation between amino acid transport and virulence was established in an antibiotic-resistant pathogenic prokaryote. In this case, the deletion of three genes encoding branched-chain amino acid (BCAA) permeases caused virulence attenuation in *Staphylococcus aureus* [71]. Our data also showed the same association, and we have further showed that the ability to invade and persist in the host, thereby causing disease, is dependent upon thermal and oxidative stress resistance. It is interesting to note that amino acid uptake must be an important feature of stress resistance, since two redundant permeases were found and only the lack of both of them led to failure in the thermal and oxidative stress response and virulence attenuation. This result suggests that Aap4 and Aap5, in fact, have overlapping functions, have high transport capacity and are specialized for transport at higher temperatures. The fact that thermosensitivity can be remedied by Na and alkaline conditions (high pH) argues that the remaining permeases are not necessarily required during thermal and oxidative stress responses, but their transport ability may be improved in the presence of ions. This result can be reconciled with the fact that all permeases found in the *C*. *neoformans* genome belong to the APC-superfamily, which is comprised of proton-driven permeases. As a consequence, it seems, the more ions available, the better is the transport efficiency.

In addition, we found that amino acid uptake is important for fluconazole resistance (Table 3). This may represent a possible synergistic use of permease inhibitors and fluconazole therapy. Along the same line, our study validated permeases Aap4 and Aap5 as the target of an antifungal agent, eugenol, a natural compound extracted from plants [72,73]. Previous work has shown that *S*. *cerevisiae* TAT2 permease is the target of eugenol [49]. These authors showed that the inhibitor does not disturb the biosynthetic pathway of aromatic and branched-chain amino acids; they suggested that its target is the amino acid permeases. Overexpression of *GAP*1 and *TAT*2 render cells hypersensitive to inhibitors, since the molecules targeted by eugenol are overproduced. In our study, *C*. *neoformans* was very sensitive to eugenol, but the deletion of permeases Aap2, Aap4 and Aap5 rendered the cell resistant to the inhibitor, thus suggesting that in *C*. *neoformans* these permeases are the targets of eugenol.

In summary, our data point out for the first time that amino acid uptake is essential for virulence in *C*. *neoformans*, since it is required for high-temperature growth, stress and antifungal resistance in this basidiomycete. Our data not only unravel important biological aspects of this yeast’s physiology regarding its nutritional requirements, but also raises the possibility of a new drug target and a specific inhibitor that could be considered as a drug prototype. Easy pharmacological access and high selective toxicity are two main criteria for a biological process to be considered an excellent drug target. Amino acid uptake by permeases fulfills these requirements, since permeases are exposed in the cell surface and are widely diverse between humans and yeasts.

## Supporting Information

S1 Fig Schematic representation of wild type and mutant *loci* and southern blot analysis of single and double mutants. (A) *aap*2Δ; (B) *aap*4Δ; (C) *aap*5Δ; (D) *aap*4Δ/*aap*5Δ; (E) *mup*1Δ; (F) *mup*3Δ and (G) *mup*1Δ/*mup*3Δ. MW = molecular weight; Kb = kilobase pairs.
(PDF)

S2 Fig Growth of H99 strain and single mutants (*aap*4Δ and *aap*5Δ, two individual mutants each) in YEPD at pH 5.2 without supplements or supplemented with NaCl (0.75 M), in YEPD at pHs 6 and 7 (without salt supplements) at 30 and 37°C. Serial dilutions represent 10^4^, 10^3^, 10^2^, 10^1^ and 1 cell.
(TIF)

S1 Table Strains list. (DOCX)

S2 Table Primers list. (DOCX)

## References

[1] Schwartzman JA, Ruby EG (2016) Stress as a Normal Cue in the Symbiotic Environment. Trends Microbiol 24: 414–424. 10.1016/j.tim.2016.02.012 27004825 PMC4841697

[2] Dantas Ada S, Day A, Ikeh M, Kos I, Achan B, Quinn J (2015) Oxidative stress responses in the human fungal pathogen, *Candida albicans*. Biomolecules 5: 142–165. 10.3390/biom5010142 25723552 PMC4384116

[3] Dichtl K, Samantaray S, Wagener J (2016) Cell wall integrity signaling in human pathogenic fungi. Cell Microbiol. 10.1111/cmi.12612 27155139

[4] Shor E, Perlin DS (2015) Coping with stress and the emergence of multidrug resistance in fungi. PLoS Pathog 11: e1004668 10.1371/journal.ppat.1004668 25790300 PMC4366371

[5] Sui Y, Wisniewski M, Droby S, Liu J (2015) Responses of yeast biocontrol agents to environmental stress. Appl Environ Microbiol 81: 2968–2975. 10.1128/AEM.04203-14 25710368 PMC4393439

[6] Kronstad J, Saikia S, Nielson ED, Kretschmer M, Jung W, Hu G, et al (2012) Adaptation of *Cryptococcus neoformans* to mammalian hosts: integrated regulation of metabolism and virulence. Eukaryot Cell 11: 109–118. 10.1128/EC.05273-11 22140231 PMC3272904

[7] Hu G, Cheng PY, Sham A, Perfect JR, Kronstad JW (2008) Metabolic adaptation in *Cryptococcus neoformans* during early murine pulmonary infection. Mol Microbiol 69: 1456–1475. 10.1111/j.1365-2958.2008.06374.x 18673460 PMC2730461

[8] Ene IV, Brunke S, Brown AJ, Hube B (2014) Metabolism in fungal pathogenesis. Cold Spring Harb Perspect Med 4: a019695 10.1101/cshperspect.a019695 25190251 PMC4292087

[9] Li SS, Mody CH (2010) Cryptococcus. Proc Am Thorac Soc 7: 186–196. 10.1513/pats.200907-063AL 20463247

[10] Lin X (2009) *Cryptococcus neoformans*: morphogenesis, infection, and evolution. Infect Genet Evol 9: 401–416. 10.1016/j.meegid.2009.01.013 19460306

[11] Lin X, Heitman J (2006) The biology of the *Cryptococcus neoformans* species complex. Annu Rev Microbiol 60: 69–105. 10.1146/annurev.micro.60.080805.142102 16704346

[12] Antinori S (2013) New Insights into HIV/AIDS-Associated Cryptococcosis. ISRN AIDS 2013: 471363 10.1155/2013/471363 24052889 PMC3767198

[13] Srikanta D, Santiago-Tirado FH, Doering TL (2014) *Cryptococcus neoformans*: historical curiosity to modern pathogen. Yeast 31: 47–60. 10.1002/yea.2997 24375706 PMC3938112

[14] Voelz K, May RC (2010) Cryptococcal interactions with the host immune system. Eukaryot Cell 9: 835–846. 10.1128/EC.00039-10 20382758 PMC2901644

[15] Liu TB, Perlin DS, Xue C (2012) Molecular mechanisms of cryptococcal meningitis. Virulence 3: 173–181. 10.4161/viru.18685 22460646 PMC3396696

[16] Park BJ, Wannemuehler Kathleen a, Marston Barbara J, Govender Nelesh, Pappas Peter G, and Chiller Tom M (2009) Estimation of the Current Global Burden of Cryptococcal Meningitis among Persons Living with HIV/AIDS. AIDS (London, England) 23: 525–530. 10.1097/QAD.0b013e328322ffac 19182676

[17] Nielsen K, Cox GM, Wang P, Toffaletti DL, Perfect JR, Heitman J (2003) Sexual cycle of *Cryptococcus neoformans* var. *grubii* and virulence of congenic a and alpha isolates. Infect Immun 71: 4831–4841. 12933823 10.1128/IAI.71.9.4831-4841.2003 PMC187335

[18] Wang P, Heitman J (1999) Signal transduction cascades regulating mating, filamentation, and virulence in *Cryptococcus neoformans*. Curr Opin Microbiol 2: 358–362. 10.1016/S1369-5274(99)80063-0 10458985

[19] Cox GM, Harrison TS, McDade HC, Taborda CP, Heinrich G, Casadevall A, et al (2003) Superoxide dismutase influences the virulence of *Cryptococcus neoformans* by affecting growth within macrophages. Infect Immun 71: 173–180. 10.1128/IAI.71.1.173-180.2003 12496163 PMC143417

[20] Doering TL (2009) How sweet it is! Cell wall biogenesis and polysaccharide capsule formation in *Cryptococcus neoformans*. Annu Rev Microbiol 63: 223–247. 10.1146/annurev.micro.62.081307.162753 19575556 PMC2880894

[21] Janbon G (2004) Cryptococcus neoformans capsule biosynthesis and regulation. FEMS Yeast Res 4: 765–771. 10.1016/j.femsyr.2004.04.003 15450183

[22] Karkowska-Kuleta J, Rapala-Kozik M, Kozik A (2009) Fungi pathogenic to humans: molecular bases of virulence of *Candida albicans*, *Cryptococcus neoformans* and *Aspergillus fumigatus*. Acta Biochim Pol 56: 211–224. 19543556

[23] Komalapriya C, Kaloriti D, Tillmann AT, Yin Z, Herrero de Dios C, Jacobsen MD, et al (2015) Integrative Model of Oxidative Stress Adaptation in the Fungal Pathogen *Candida albicans*. PLoS One 10: e0137750 10.1371/journal.pone.0137750 26368573 PMC4569071

[24] Kretschmer M, Reiner E, Hu G, Tam N, Oliveira DL, Caza M, et al (2014) Defects in phosphate acquisition and storage influence virulence of *Cryptococcus neoformans*. Infect Immun 82: 2697–2712. 10.1128/IAI.01607-14 24711572 PMC4097646

[25] Lee IR, Chow EW, Morrow CA, Djordjevic JT, Fraser JA (2011) Nitrogen metabolite repression of metabolism and virulence in the human fungal pathogen *Cryptococcus neoformans*. Genetics 188: 309–323. 10.1534/genetics.111.128538 21441208 PMC3122321

[26] Ost KS, O'Meara TR, Huda N, Esher SK, Alspaugh JA (2015) The *Cryptococcus neoformans* alkaline response pathway: identification of a novel rim pathway activator. PLoS Genet 11: e1005159 10.1371/journal.pgen.1005159 25859664 PMC4393102

[27] Fernandes JD, Martho K, Tofik V, Vallim MA, Pascon RC (2015) The Role of Amino Acid Permeases and Tryptophan Biosynthesis in *Cryptococcus neoformans* Survival. PLoS One 10: e0132369 10.1371/journal.pone.0132369 26162077 PMC4498599

[28] Kingsbury JM, McCusker JH (2008) Threonine biosynthetic genes are essential in *Cryptococcus neoformans*. Microbiology 154: 2767–2775. 10.1099/mic.0.2008/019729-0 18757810 PMC2674386

[29] Kingsbury JM, McCusker JH (2010) Fungal homoserine kinase (thr1Delta) mutants are attenuated in virulence and die rapidly upon threonine starvation and serum incubation. Eukaryot Cell 9: 729–737. 10.1128/EC.00045-10 20305003 PMC2863962

[30] Kingsbury JM, McCusker JH (2010) Cytocidal amino acid starvation of *Saccharomyces cerevisiae* and *Candida albicans* acetolactate synthase (ilv2{Delta}) mutants is influenced by the carbon source and rapamycin. Microbiology 156: 929–939. 10.1099/mic.0.034348-0 20019084 PMC2841795

[31] Kingsbury JM, Yang Z, Ganous TM, Cox GM, McCusker JH (2004) *Cryptococcus neoformans* Ilv2p confers resistance to sulfometuron methyl and is required for survival at 37 degrees C and in vivo. Microbiology 150: 1547–1558. 10.1099/mic.0.26928-0 15133116

[32] Kingsbury JM, Yang Z, Ganous TM, Cox GM, McCusker JH (2004) Novel chimeric spermidine synthase-saccharopine dehydrogenase gene (SPE3-LYS9) in the human pathogen *Cryptococcus neoformans*. Eukaryot Cell 3: 752–763. 10.1128/EC.3.3.752-763.2004 15189996 PMC420128

[33] Pascon RC, Ganous TM, Kingsbury JM, Cox GM, McCusker JH (2004) *Cryptococcus neoformans* methionine synthase: expression analysis and requirement for virulence. Microbiology 150: 3013–3023. 10.1099/mic.0.27235-0 15347759

[34] Yang Z, Pascon RC, Alspaugh A, Cox GM, McCusker JH (2002) Molecular and genetic analysis of the *Cryptococcus neoformans* MET3 gene and a met3 mutant. Microbiology 148: 2617–2625. 10.1099/00221287-148-8-2617 12177356

[35] Saier MH Jr. (2000) Families of transmembrane transporters selective for amino acids and their derivatives. Microbiology 146 (Pt 8): 1775–1795. 10.1099/00221287-146-8-1775 10931885

[36] Schweikhard ES, Ziegler CM (2012) Amino acid secondary transporters: toward a common transport mechanism. Curr Top Membr 70: 1–28. 10.1016/B978-0-12-394316-3.00001-6 23177982

[37] Paulsen IT, Sliwinski MK, Nelissen B, Goffeau A, Saier MH Jr. (1998) Unified inventory of established and putative transporters encoded within the complete genome of *Saccharomyces cerevisiae*. FEBS Lett 430: 116–125. 9678606 10.1016/s0014-5793(98)00629-2

[38] Ljungdahl PO, Daignan-Fornier B (2012) Regulation of amino acid, nucleotide, and phosphate metabolism in *Saccharomyces cerevisiae*. Genetics 190: 885–929. 10.1534/genetics.111.133306 22419079 PMC3296254

[39] Wipf D, Ludewig U, Tegeder M, Rentsch D, Koch W, Frommer WB (2002) Conservation of amino acid transporters in fungi, plants and animals. Trends Biochem Sci 27: 139–147. 10.1016/S0968-0004(01)02054-0 11893511

[40] Regenberg B, During-Olsen L, Kielland-Brandt MC, Holmberg S (1999) Substrate specificity and gene expression of the amino-acid permeases in *Saccharomyces cerevisiae*. Curr Genet 36: 317–328. 10.1007/s002940050506 10654085

[41] Ljungdahl PO (2009) Amino-acid-induced signalling via the SPS-sensing pathway in yeast. Biochem Soc Trans 37: 242–247. 10.1042/BST0370242 19143640

[42] Magasanik B, Kaiser CA (2002) Nitrogen regulation in *Saccharomyces cerevisiae*. Gene 290: 1–18. 10.1016/S0378-1119(02)00558-9 12062797

[43] Kraidlova L, Van Zeebroeck G, Van Dijck P, Sychrova H (2011) The *Candida albicans* GAP gene family encodes permeases involved in general and specific amino acid uptake and sensing. Eukaryot Cell 10: 1219–1229. 10.1128/EC.05026-11 21764911 PMC3187050

[44] Biswas K, Rieger KJ, Morschhauser J (2003) Functional characterization of CaCBF1, the *Candida albicans* homolog of centromere binding factor 1. Gene 323: 43–55. 10.1016/j.gene.2003.09.005 14659878

[45] Brega E, Zufferey R, Mamoun CB (2004) *Candida albicans* Csy1p is a nutrient sensor important for activation of amino acid uptake and hyphal morphogenesis. Eukaryot Cell 3: 135–143. 10.1128/EC.3.1.135-143.2004 14871944 PMC329513

[46] Martinez P, Ljungdahl PO (2004) An ER packaging chaperone determines the amino acid uptake capacity and virulence of *Candida albicans*. Mol Microbiol 51: 371–384. 10.1046/j.1365-2958.2003.03845.x 14756779

[47] Mathieu C, Gonzalez Salgado A, Wirdnam C, Meier S, Grotemeyer MS, Inbar E, et al (2014) *Trypanosoma brucei* eflornithine transporter AAT6 is a low-affinity low-selective transporter for neutral amino acids. Biochem J 463: 9–18. 10.1042/BJ20140719 24988048

[48] Saye M, Miranda MR, di Girolamo F, de los Milagros Camara M, Pereira CA (2014) Proline modulates the *Trypanosoma cruzi* resistance to reactive oxygen species and drugs through a novel D, L-proline transporter. PLoS One 9: e92028 10.1371/journal.pone.0092028 24637744 PMC3956872

[49] Darvishi E, Omidi M, Bushehri AA, Golshani A, Smith ML (2013) The antifungal eugenol perturbs dual aromatic and branched-chain amino acid permeases in the cytoplasmic membrane of yeast. PLoS One 8: e76028 10.1371/journal.pone.0076028 24204588 PMC3799837

[50] Kim MS, Kim SY, Jung KW, Bahn YS (2012) Targeted gene disruption in *Cryptococcus neoformans* using double-joint PCR with split dominant selectable markers. Methods Mol Biol 845: 67–84. 10.1007/978-1-61779-539-8_5 22328368

[51] Toffaletti DL, Rude TH, Johnston SA, Durack DT, Perfect JR (1993) Gene transfer in *Cryptococcus neoformans* by use of biolistic delivery of DNA. J Bacteriol 175: 1405–1411. 8444802 10.1128/jb.175.5.1405-1411.1993 PMC193227

[52] O'Meara TR, Xu W, Selvig KM, O'Meara MJ, Mitchell AP, Alspaugh JA (2014) The *Cryptococcus neoformans* Rim101 transcription factor directly regulates genes required for adaptation to the host. Mol Cell Biol 34: 673–684. 10.1128/MCB.01359-13 24324006 PMC3911494

[53] Zaragoza O, Fries BC, Casadevall A (2003) Induction of capsule growth in *Cryptococcus neoformans* by mammalian serum and CO(2). Infect Immun 71: 6155–6164. 14573631 10.1128/IAI.71.11.6155-6164.2003 PMC219591

[54] Christensen WB (1946) Urea Decomposition as a Means of Differentiating Proteus and Paracolon Cultures from Each Other and from *Salmonella* and *Shigella* Types. J Bacteriol 52: 461–466. 16561200 10.1128/jb.52.4.461-466.1946 PMC518212

[55] Berger S, Sicker D (2009) Classics in Spectroscopy. Isolation and Strucutral elucidation of Natural products.

[56] Mylonakis E, Moreno R, El Khoury JB, Idnurm A, Heitman J, Calderwood SB, et al (2005) Galleria mellonella as a model system to study *Cryptococcus neoformans* pathogenesis. Infect Immun 73: 3842–3850. 10.1128/IAI.73.7.3842-3850.2005 15972469 PMC1168598

[57] Skowyra ML, Doering TL (2012) RNA interference in *Cryptococcus neoformans*. Methods Mol Biol 845: 165–186. 10.1007/978-1-61779-539-8_11 22328374 PMC3708647

[58] Livak KJ, Schmittgen TD (2001) Analysis of relative gene expression data using real-time quantitative PCR and the 2(-Delta Delta C(T)) Method. Methods 25: 402–408. 10.1006/meth.2001.1262 11846609

[59] Alspaugh JA, Cavallo LM, Perfect JR, Heitman J (2000) RAS1 regulates filamentation, mating and growth at high temperature of *Cryptococcus neoformans*. Mol Microbiol 36: 352–365. 10.1046/j.1365-2958.2000.01852.x 10792722

[60] Alspaugh JA, Perfect JR, Heitman J (1998) Signal transduction pathways regulating differentiation and pathogenicity of *Cryptococcus neoformans*. Fungal Genet Biol 25: 1–14. 10.1006/fgbi.1998.1079 9806801

[61] Ballou ER, Kozubowski L, Nichols CB, Alspaugh JA (2013) Ras1 acts through duplicated Cdc42 and Rac proteins to regulate morphogenesis and pathogenesis in the human fungal pathogen *Cryptococcus neoformans*. PLoS Genet 9: e1003687 10.1371/journal.pgen.1003687 23950731 PMC3738472

[62] Nichols CB, Ost KS, Grogan DP, Pianalto K, Hasan S, Alspaugh JA (2015) Impact of Protein Palmitoylation on the Virulence Potential of *Cryptococcus neoformans*. Eukaryot Cell 14: 626–635. 10.1128/EC.00010-15 25862155 PMC4486677

[63] Chang YC, Khanal Lamichhane A, Bradley J, Rodgers L, Ngamskulrungroj P, Kwon-Chung KJ (2015) Differences between *Cryptococcus neoformans* and *Cryptococcus gattii* in the Molecular Mechanisms Governing Utilization of D-Amino Acids as the Sole Nitrogen Source. PLoS One 10: e0131865 10.1371/journal.pone.0131865 26132227 PMC4489021

[64] Do E, Park M, Hu G, Caza M, Kronstad JW, Jung WH (2016) The lysine biosynthetic enzyme Lys4 influences iron metabolism, mitochondrial function and virulence in *Cryptococcus neoformans*. Biochemical and Biophysical Research Communications In press. 10.1016/j.bbrc.2016.06.123 27353379 PMC5183541

[65] Kosugi A, Koizumi Y, Yanagida F, Udaka S (2001) MUP1, high affinity methionine permease, is involved in cysteine uptake by *Saccharomyces cerevisiae*. Biosci Biotechnol Biochem 65: 728–731. 10.1271/bbb.65.728 11330701

[66] Isnard AD, Thomas D, Surdin-Kerjan Y (1996) The study of methionine uptake in *Saccharomyces cerevisiae* reveals a new family of amino acid permeases. J Mol Biol 262: 473–484. 10.1006/jmbi.1996.0529 8893857

[67] Grenson M, Hou C, Crabeel M (1970) Multiplicity of the amino acid permeases in *Saccharomyces cerevisiae*. IV. Evidence for a general amino acid permease. J Bacteriol 103: 770–777. 5474888 10.1128/jb.103.3.770-777.1970 PMC248157

[68] Liu TB, Wang Y, Baker GM, Fahmy H, Jiang L, Xue C (2013) The glucose sensor-like protein Hxs1 is a high-affinity glucose transporter and required for virulence in *Cryptococcus neoformans*. PLoS One 8: e64239 10.1371/journal.pone.0064239 23691177 PMC3653957

[69] Han K, Do E, Jung WH (2012) A human fungal pathogen *Cryptococcus neoformans* expresses three distinct iron permease homologs. J Microbiol Biotechnol 22: 1644–1652. 10.4014/jmb.1209.09019 23221526

[70] Smith DG, Garcia-Pedrajas MD, Gold SE, Perlin MH (2003) Isolation and characterization from pathogenic fungi of genes encoding ammonium permeases and their roles in dimorphism. Mol Microbiol 50: 259–275. 10.1046/j.1365-2958.2003.03680.x 14507379

[71] Kaiser JC, Omer S, Sheldon JR, Welch I, Heinrichs DE (2015) Role of BrnQ1 and BrnQ2 in branched-chain amino acid transport and virulence in *Staphylococcus aureus*. Infect Immun 83: 1019–1029. 10.1128/IAI.02542-14 25547798 PMC4333469

[72] Kamatou GP, Vermaak I, Viljoen AM (2012) Eugenol—from the remote Maluku Islands to the international market place: a review of a remarkable and versatile molecule. Molecules 17: 6953–6981. 10.3390/molecules17066953 22728369 PMC6268661

[73] Pramod K, Ansari SH, Ali J (2010) Eugenol: a natural compound with versatile pharmacological actions. Nat Prod Commun 5: 1999–2006. 21299140

