# Supplementary material for: Amino Acid Permeases and Virulence in Cryptococcus neoformans
Source: PLoS One. 2016 Oct 3;11(10):e0163919. doi: 10.1371/journal.pone.0163919 (PMC5047642; doi:10.1371/journal.pone.0163919)
Supplement: S1 Fig — (A) aap2Δ; (B) aap4Δ; (C) aap5Δ; (D) aap4Δ/aap5Δ; (E) mup1Δ; (F) mup3Δ and (G) mup1Δ/mup3Δ. MW = molecular weight; Kb = kilobase pairs. (PDF) [file pone.0163919.s001.pdf]

**(A)**

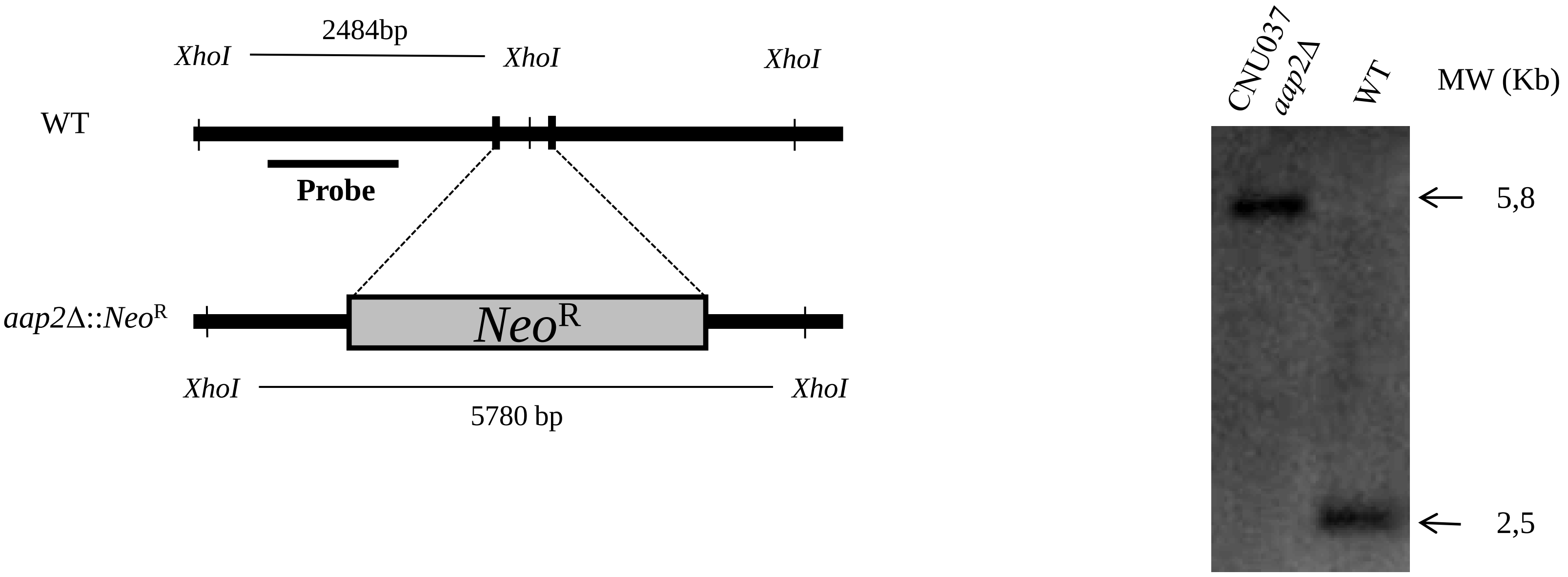


**S1 Fig.** Schematic representation of wild type and mutant loci and s*outhern blot* analysis of single and double mutants. **(A)** *aap*2Δ; (B) *aap*4Δ; (C) *aap*5Δ; (D*) aap*4Δ/*aap*5Δ; (E) *mup*1Δ; (F) *mup*3Δ and (G) *mup*1Δ /*mup*3Δ. MW = molecular weight; Kb = kilobase pairs

**(B)**

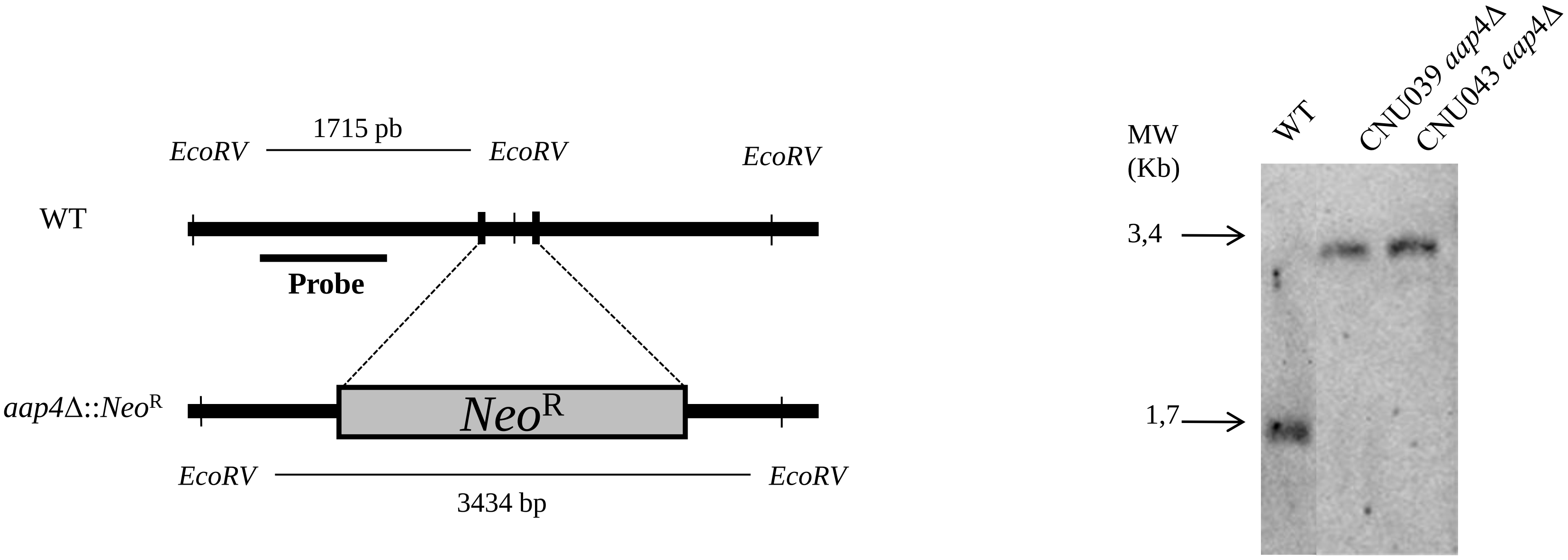


**Supplementary Figure 1.** S*outhern blot* analysis of *aap*4Δ::*Neo*$^{R}$. **(C)** Schematic representation of *AAP*4 *locus* with *EcoRV* sites. **(D)** Restriction pattern of *AAP4 locus* in the wild type (WT) H99 and mutant strains (CNU039 and 043). MW = molecular weight; Kb = kilobase pairs

**(C)**

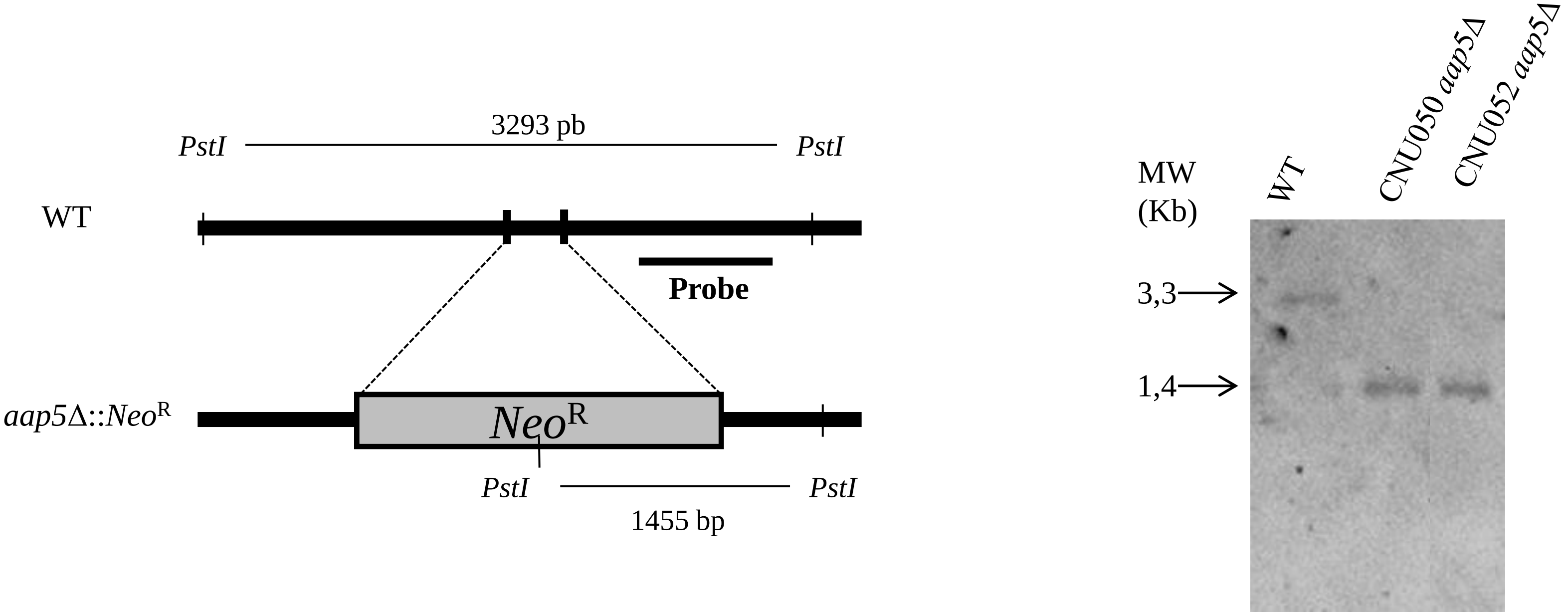


**Supplementary Figure 1.** S*outhern blot* analysis of *aap*5Δ::*Neo*R. **(E)** Schematic representation of *AAP*5 *locus* with *PstI* sites. **(F)** Restriction pattern of *AAP5 locus* in the wild type (WT) H99 and mutant strains (CNU050 and 052). MW = molecular weight; Kb = kilobase pairs

**(D)**

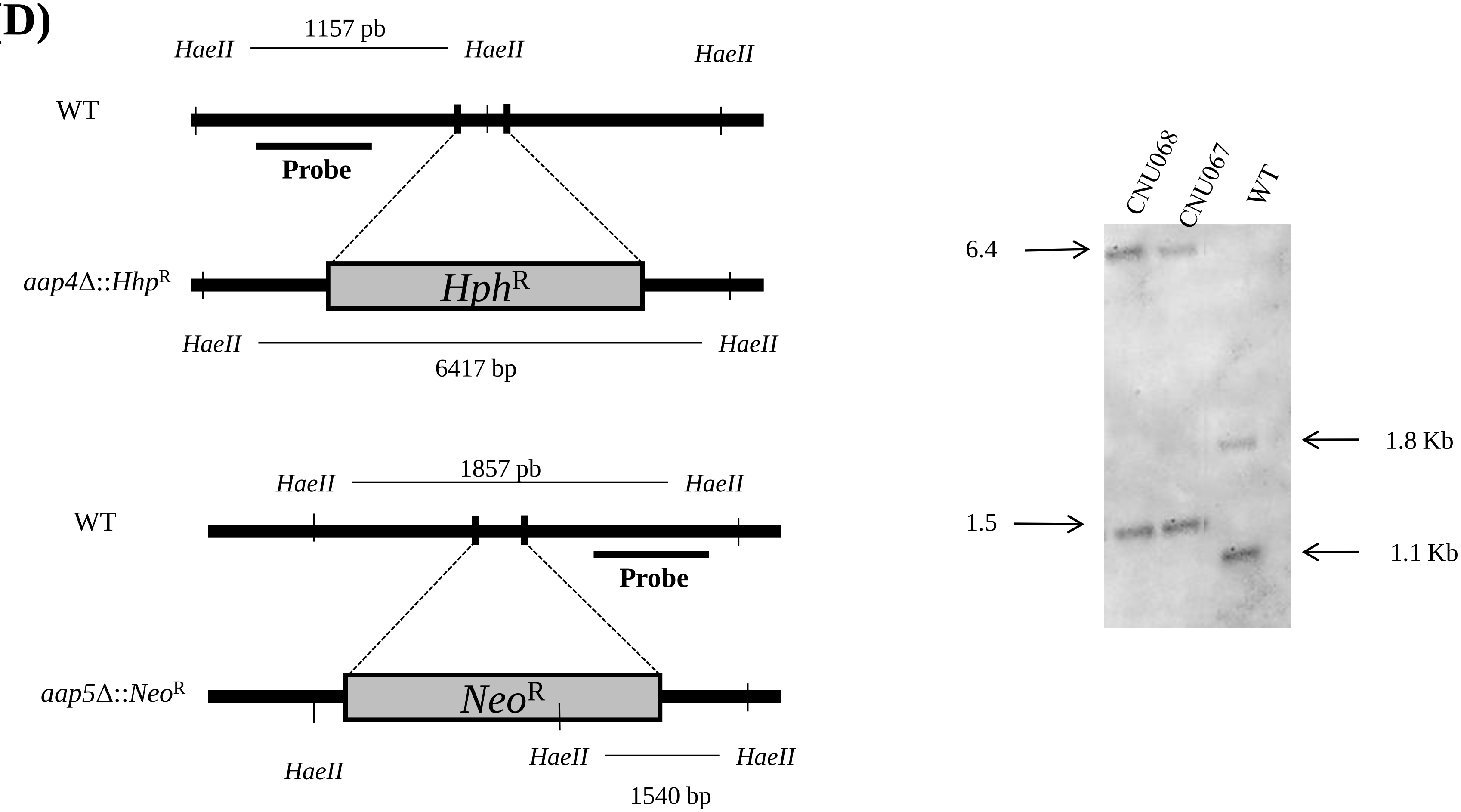


**Supplementary Figure 1.** S*outhern blot* analysis of *aap4*Δ::*Hph*$^{R}$/*aap*5Δ::*Neo*$^{R}$. **(G)** Schematic representation of *AAP*4 and *AAP*5 *locus* with *HaeII* sites. **(H)** Restriction pattern of *AAP4 and AAP5 locus* in the wild type (WT) H99 and mutant strains (CNU067 and 068). MW = molecular weight; Kb = kilobase pairs

# (E)

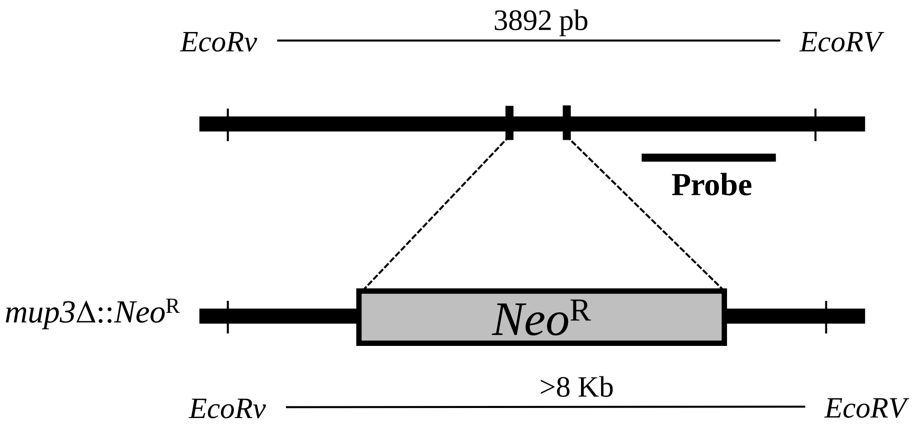


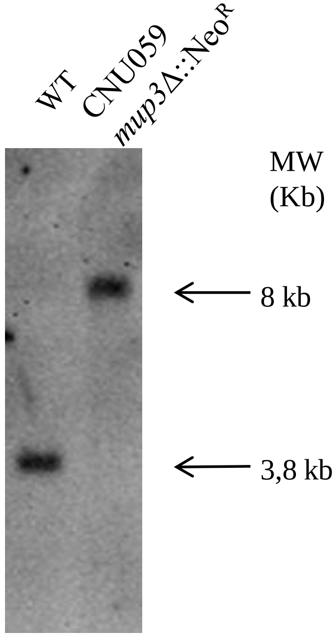


**Supplementary Figure 1.** S*outhern blot* analysis of *mup3*Δ::*Neo*$^{R}$. **(E)** Schematic representation of *MUP3 locus* with *EcoRV* sites. **(F)** Restriction pattern of *MUP3 locus* in the wild type (WT) H99 and mutant strain (CNU0059). MW = molecular weight; Kb = kilobase pairs

**(F)**

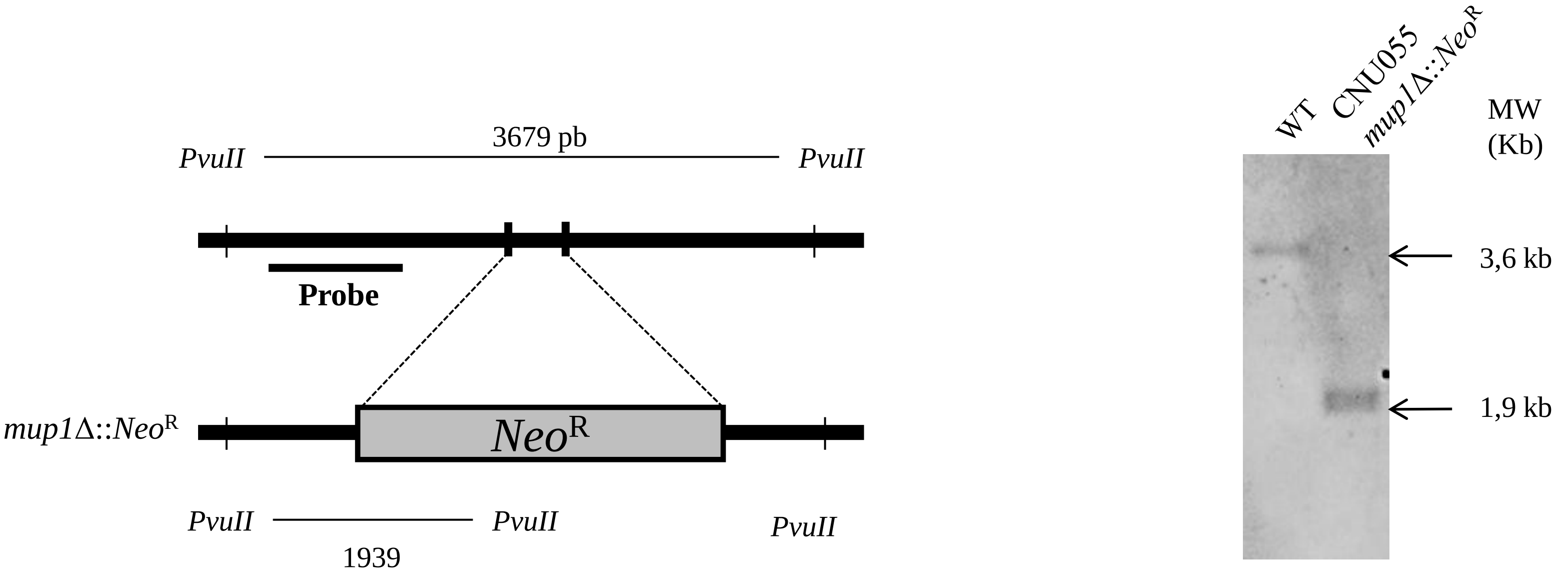


**Supplementary Figure 1.** S*outhern blot* analysis of *mup1*Δ::*Neo*$^{R}$. **(E)** Schematic representation of *MUP1 locus* with *PvuII* sites. **(F)** Restriction pattern of *MUP1 locus* in the wild type (WT) H99 and mutant strain (CNU0055). MW = molecular weight; Kb = kilobase pairs

(G)

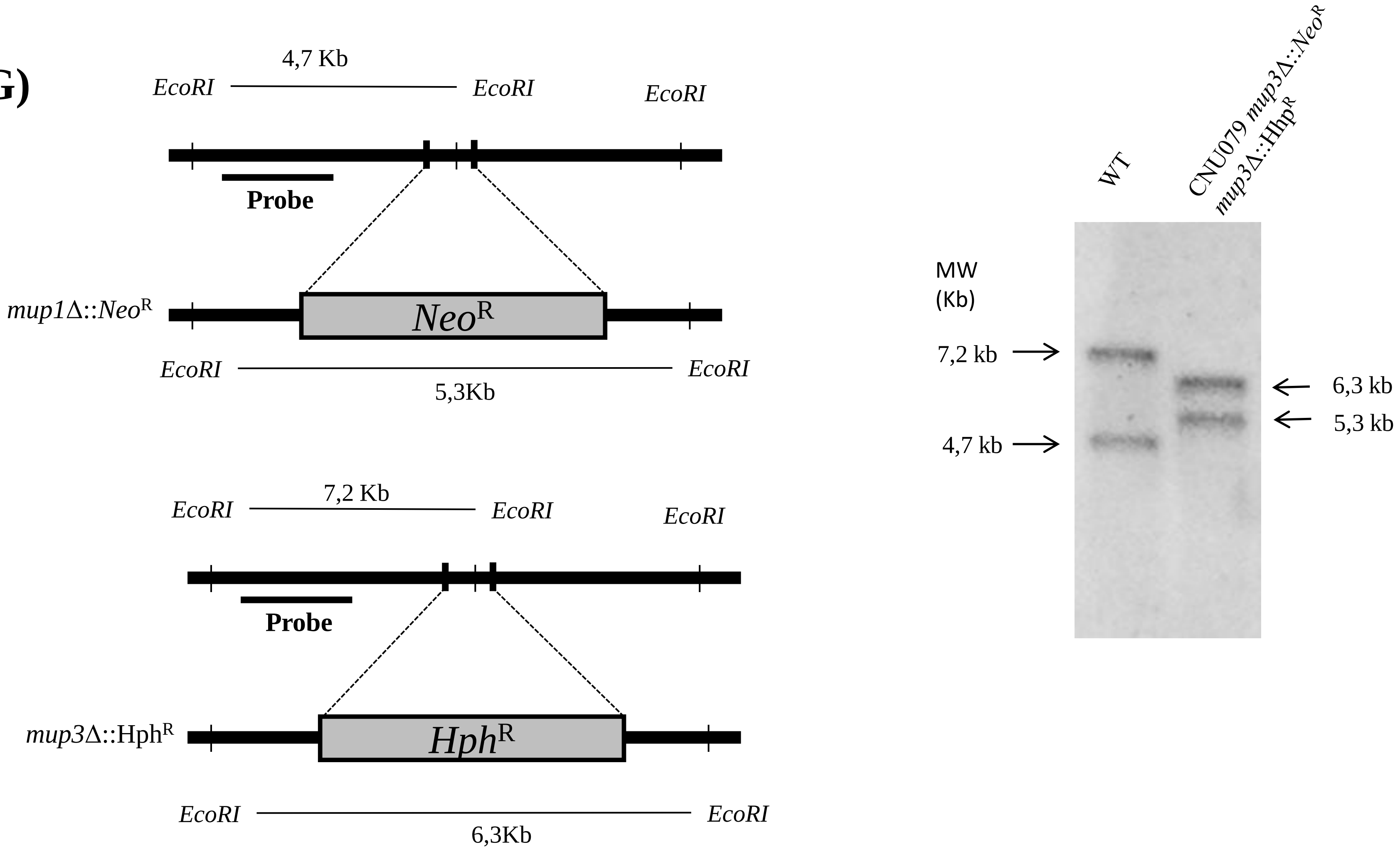


**Supplementary Figure 1.** S*outhern blot* analysis of *mup1*Δ::*Neo*R; *mup3*Δ::*Hhp*R. **(M)** Schematic representation of *MUP1* and *MUP*3 *locus* with *EcoRI* sites. **(N)** Restriction pattern of *MUP1 and MUP3 locus* in the wild type (WT) H99 and mutant strain (CNU079). MW = molecular weight; Kb = kilobase pairs
