# Supplementary figures and images for: Amino Acid Permeases and Virulence in Cryptococcus neoformans
Source: PLoS One. 2016 Oct 3;11(10):e0163919. doi: 10.1371/journal.pone.0163919 (PMC5047642; doi:10.1371/journal.pone.0163919)

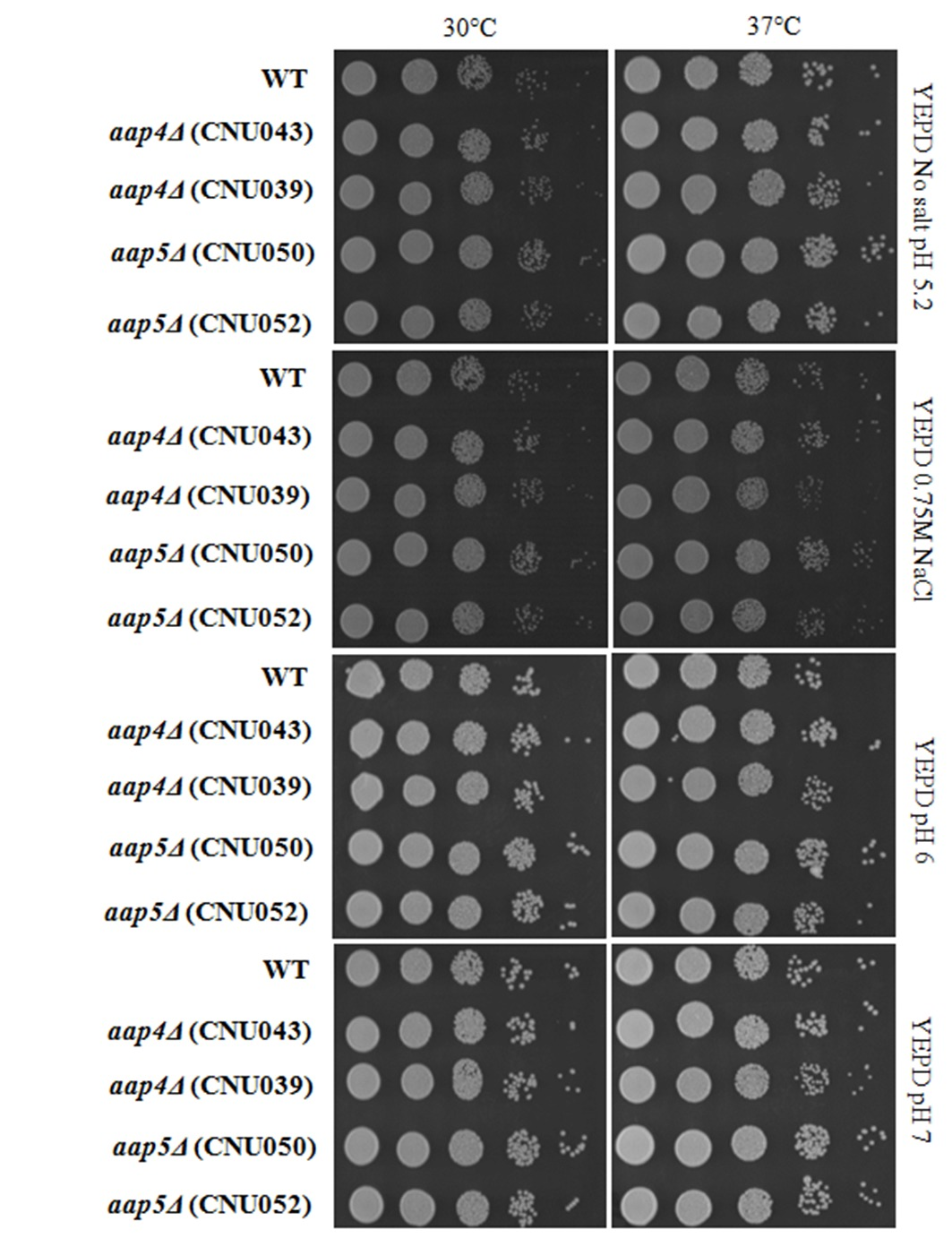

Supplement: S2 Fig — Serial dilutions represent 104, 103, 102, 101 and 1 cell. (TIF) [file pone.0163919.s002.tif]
