# Supplementary material for: Amino Acid Permeases and Virulence in Cryptococcus neoformans
Source: PLoS One. 2016 Oct 3;11(10):e0163919. doi: 10.1371/journal.pone.0163919 (PMC5047642; doi:10.1371/journal.pone.0163919)
Supplement: S1 Table — (DOCX) [file pone.0163919.s003.docx]

Table S1. Strains list

| Strain | Genotype | Reference or source |
| --- | --- | --- |
| H99 | MATα wild-type | Duke University Medical Center |
| H86 | *lac1*Δ | LIMic Unifesp |
| CNU037 | *aap2*Δ::*Neo*^R^ | This study |
| CNU039 | *aap4*Δ::*Neo*^R^ | This study |
| CNU043 | *aap4*Δ::*Neo*^R^ | This study |
| CNU050 | *aap5*Δ::*Neo*^R^ | This study |
| CNU052 | *aap5*Δ::*Neo*^R^ | This study |
| CNU067 | *aap4*Δ*::* *Hph*^R^ /*aap5*Δ::*Neo*^R^ | This study |
| CNU068 | *aap4*Δ*::* *Hph*^R^ /*aap5*Δ::*Neo*^R^ | This study |
| CNU055 | *mup1*Δ::*Neo*^R^ | This study |
| CNU059 | *mup3*Δ::*Neo*^R^ | This study |
| CNU079 | *mup1*Δ*::* *Neo*^R^/*mup3*::*Hph*^R^ | This study |
| FGC003 | *ura4*Δ::*Neo*^R^ | Gontijo *et al*., 2014 |
| BY4742 (*Saccharomyces cerevisiae*) | *MATα;his3Δ1,leu2Δ0, lys2Δ0, ura3Δ0* | Genetic Research |
