# Supplementary material for: Amino Acid Permeases and Virulence in Cryptococcus neoformans
Source: PLoS One. 2016 Oct 3;11(10):e0163919. doi: 10.1371/journal.pone.0163919 (PMC5047642; doi:10.1371/journal.pone.0163919)
Supplement: S2 Table — (DOCX) [file pone.0163919.s004.docx]

Table S2. Primers list

| **Code** | **Sequence (5’ 🡪 3’)** | **F/R** | **Use** |
| --- | --- | --- | --- |
| PRCP211 | CCCGAACATCGCCTCGCTC | R | Double joint PCR Hyg |
| PRCP212 | ATCCCCATGTGTATCACTGGC | F | Double joint PCR Hyg |
| PRCP213 | ACGACGGGCGTTCCTTGCG | F | Double joint PCR Neo |
| PRCP214 | TCGCTTGGTGGTCGAATGGG | R | Double joint PCR Neo |
| PRCP252 | CAGGAATGAGGAGGGGAACC | F | Double joint left arm AAP2 |
| PRCP253 | AATGCGAAGCCGCACCACCG | R | Double joint left arm AAP2 |
| PRCP230 | CAATTCATTCACTTCCTCTAGAAGGACTGCGAGGATGTGAGCTGGAG | F | Double joint Marker AAP2 |
| PRCP231 | CAACTAACTATGATTTCTAGAAGAGATGTAGAAACTAGCTTCC | R | Double joint Marker AAP2 |
| PRCP254 | CTCCAGCTCACATCCTCGCAGTCCTTCTAGAGGAAGTGAATGAATTG | F | Double joint right arm AAP2 |
| PRCP255 | GGAAGCTAGTTTCTACATCTCTTCTAGAAATCATAGTTAGTTG | R | Double joint right arm AAP2 |
| PRCP234 | TTGAAATGAATATAAAATACA | F | Double joint left arm AAP4 |
| PRCP235 | CCAGCTCACATCCTCGCAGTCTTGATGGTTGGGTTTACAAA | R | Double joint left arm AAP4 |
| PRCP236 | TTGTAAACCCAACCATCAAGACTGCGAGGATGTGAGCTGGA | F | Double joint Marker AAP4 |
| PRCP237 | AAAATGCCAAGTATTTGAAGAGATGTAGAAACTAGCTTCCT | R | Double joint Marker AAP4 |
| PRCP238 | AGGAAGCTAGTTTCTACATCTCTTCAAATACTTGGCATTTT | F | Double joint right arm AAP4 |
| PRCP239 | GCTGAACCCTGATATCAGACT | R | Double joint right arm AAP4 |
| PRCP281 | CAAAAGCTCTTGTCTGTGACAAGAGAGGGGATGCGAGGATG | F | Double joint left arm AAP5 |
| PRCP241 | CTCCAGCTCACATCCTCGCATCCCCTCTCTTGTCACAGACAA | R | Double joint left arm AAP5 |
| PRCP242 | TTGTCTGTGACAAGAGAGGGGATGCGAGGATGTGAGCTGGAG | F | Double joint Marker AAP5 |
| PRCP243 | TTCGGATCACTACATAGATTCGGAAGAGATGTAGAAACTA | R | Double joint Marker AAP5 |
| PRCP244 | TAGTTTCTACATCTCTTCCGAATCTATGTAGTGATCCGAA | F | Double joint right arm AAP5 |
| PRCP245 | CCACGGAAACAATAAGAAGC | R | Double joint right arm AAP5 |
| PRCP265 | GTCAATTCGGAGCTGTAGAAC | F | Double joint left arm MUP3 |
| PRCP266 | CCATAATGCAATAGAGCCCCTGCGAGGATGTGAGCTGGAG | R | Double joint left arm MUP3 |
| PRCP267 | CTCCAGCTCACATCCTCGCAGGGGCTCTATTGCATTATGG | F | Double joint Marker MUP3 |
| PRCP268 | GGAAGCTAGTTTCTACATCTCTTCCTTCGATTTTAGCTATATC | R | Double joint Marker MUP3 |
| PRCP269 | GATATAGCTAAAATCGAAGGAAGAGATGTAGAAACTAGCTTCC | F | Double joint right arm MUP3 |
| PRCP270 | TCTCAAGGCTAACAATTTGATGC | R | Double joint right arm MUP3 |
| PRCP272 | CTCTGCCTGGTTAATGTGCTTC | F | Double joint left arm MUP1 |
| PRCP273 | CTCCAGCTCACATCCTCGCATGTTGTTACCTGTCGATTAG | R | Double joint left arm MUP1 |
| PRCP274 | CTAATCGACAGGTAACAACATGCGAGGATGTGAGCTGGAG | F | Double joint Marker MUP1 |
| PRCP275 | GGAACACGACATCTAAATCCGAAGAGATGTAGAAACTAGCTTCC | R | Double joint Marker MUP1 |
| PRCP276 | GGAAGCTAGTTTCTACATCTCTTCGGATTTAGATGTCGTGTTCC | F | Double joint right arm MUP1 |
| PRCP277 | CCGCTGAACGAATCATTAGAC | R | Double joint right arm MUP1 |
| PRCP278 | CCTTGTGATGGCGGATGCCC | F | Diagnostic PCR AAP2 |
| PRCP279 | GGAGGAGTGGAGCGTCGATC | F | Diagnostic PCR AAP4 |
| PRCP280 | GGCAGGAATGAGGAGGGGAAC | F | Diagnostic PCR AAP5 |
| PRCP264 | CAATACCGTTGGCTCTTCC | F | Diagnostic PCR MUP3 |
| PRCP271 | CAAGCTTGCGGTTTGAAGG | F | Diagnostic PCR MUP1 |
| PRCP187 | CATGGTATACGCGATGATGG | F | RT-PCR AAP2 |
| PRCP188 | TCTGGCTCCCCAGAAGTTAATG | R | RT-PCR AAP2 |
| PRCP191 | CGAGGCAAAGAACCCACG | F | RT-PCR AAP4 |
| PRCP192 | AATCAAGATGCAAGCGTTTATG | R | RT-PCR AAP4 |
| PRCP193 | ACTTACTTGGACCTCTATCCTC | F | RT-PCR AAP5 |
| PRCP194 | TTTTCGGATCAGCTTGAAACC | R | RT-PCR AAP5 |
| PRCP284 | GTTCAACTTCATTGTTGCCC | F | RT-PCR MUP1 |
| PRCP285 | AGGAACACGATCCCTAGTC | R | RT-PCR MUP1 |
| PRCP286 | TATGTGGCAGGCGAAATGC | F | RT-PCR MUP3 |
| PRCP287 | CCTTGCAGTTGTGTACAATC | R | RT-PCR MUP3 |
